# Transcatheter Intra‐Arterial Delivery of a Platelet‐Derived Extracellular Vesicle‐Enriched Preparation for Attenuating Skeletal Muscle Ischaemia‐Reperfusion Injury in a Rodent Forelimb Model

**DOI:** 10.1002/jev2.70247

**Published:** 2026-04-17

**Authors:** Omar A. Selim, Aida Sarcon, Atta Behfar, Chunfeng Zhao, Steven L. Moran

**Affiliations:** ^1^ Department of Orthopedic Surgery Mayo Clinic Rochester Minnesota USA; ^2^ T32 Musculoskeletal Research Training Program Mayo Clinic Rochester Minnesota USA; ^3^ Division of Plastic Surgery Mayo Clinic Rochester Minnesota USA; ^4^ Van Cleve Cardiac Regenerative Medicine Program, Center for Regenerative Medicine Mato Clinic Rochester Minnesota USA; ^5^ Department of Cardiovascular Medicine Mayo Clinic Rochester Minnesota USA; ^6^ Department of Molecular Pharmacology and Experimental Therapeutics Mayo Clinic Rochester Minnesota USA

**Keywords:** cytokine storm, extracellular vesicles, limb preservation, platelet‐derived EVs, rhabdomyolysis, skeletal muscle ischaemia‐reperfusion injury, transcatheter extremity infusion, peripheral limb ischemia, isolated limb perfusion, exosomes

## Abstract

Extracellular vesicles (EVs) are non‐replicating, lipid membrane‐bound nanoparticles released by most eukaryotic cells into the extracellular space. When derived from platelets, a subset of EVs can exhibit remarkable anti‐inflammatory and antioxidant properties, making them attractive candidates to attenuate sterile inflammatory states such as limb skeletal muscle ischaemia‐reperfusion injury (IRI). Peripheral extremity IRI complicating acute limb revascularisation procedures, replantation of major traumatic amputations and limb allotransplants can trigger a life‐threatening reperfusion syndrome that leads to multi‐organ system failure and death. In this study, we used a platelet‐derived EVs‐enriched preparation as a therapeutic strategy for extremity IRI. To allow for a clinically translatable drug delivery strategy, we established and validated a catheter‐directed regional intra‐arterial limb infusion (RLI) delivery approach that permitted selective and effective targeting of pEVs to limb skeletal muscles and soft tissues in the vascularly isolated rodent extremity simulating pre‐reperfusion preservation and revascularisation conditions of the ischaemic or amputated extremity. In addition to being safe and feasible, we demonstrated robust retention of EVs by skeletal muscles of the operative limb during the early critical phase of reperfusion injury following RLI. We also show that pretreatment of ischaemic skeletal muscles using an pEVs‐enriched formulation just before reestablishing extremity blood flow alleviated metabolic derangements and suppressed the systemic and localised proinflammatory cytokine response associated with skeletal muscle reperfusion and reduced histologic markers of myofibre injury without adverse effects on serum chemistries. This work opens a new perspective to investigate novel approaches for regional delivery of therapeutic pEVs to skeletal muscle to mitigate limb IRI in vivo, and to achieve both in situ and ex vivo targeted delivery of nanocarriers to the isolated limb tissue for other therapeutic applications.

## 1 Introduction

Skeletal muscles are susceptible to ischaemia‐reperfusion injury (IRI) due to their inherently high metabolic activity (Kuroda et al. 2020; Barnig et al. 2022). This type of injury is commonly encountered in vascular surgery procedures including acute limb revascularisation and abdominal aortic aneurysm repair, in orthopaedic surgery due to prolonged tourniquet use and crush syndrome, and in replantation of major traumatic amputations and vascularised composite tissue allografting (Barnig et al. 2022; Carroll and Esclamado 2000; Haruta et al. 2022). Although limb reperfusion is the definitive therapy to salvage ischaemic tissues from necrosis and normalise acidotic states, re‐establishing oxygenated blood flow to hypoxic skeletal muscles paradoxically exacerbates the initial ischaemic insult. During this phase, massive and uncontrolled burst of reactive oxygen species (ROS) occurs at the dysfunctional mitochondrial redox centres triggering an intense local and systemic inflammatory response that can increase the incidence of multiple organ failure and death (Quinlan et al. 2013).

Current IRI management paradigms focus on addressing complications following their occurrence once revascularisation is completed. These include both surgical and medical options such as regional cooling, fasciotomy, haemodialysis, correction of metabolic derangements, plasmapheresis, administration of anti‐inflammatory agents, hyperbaric oxygen, etc. (Urso 2013; Defraigne and Pincemail 1998; Apichartpiyakul et al. 2022; Simon et al. 2018; Kim et al. 2024; Oley et al. 2020). However, limited strategies exist to prevent peripheral extremity IRI, and these are only feasible in controlled clinical settings whereby pre‐reperfusion limb conditioning can be performed via intra‐arterial (IA) infusion of cytoprotective agents (CPAs) before limb revascularisation is established (Amin et al. 2018; Meyers et al. 2023; Rezaei et al. 2022).

Platelet‐derived extracellular vesicles (EVs) (pEVs) are promising cell‐free candidates for use as CPAs to attenuate the peripheral extremity post‐reperfusion syndrome due to their potent anti‐inflammatory and antioxidant properties and ease of obtention. However, there is limited knowledge of how to effectively deliver EVs to the peripheral extremity and target the different tissue types collectively and achieve locoregional selectivity. Several EVs biodistribution studies following systemic intravenous (IV) administration of nanoscale vehicles such as synthetic nanoparticles and EVs show preferential accumulation in organs of the reticuloendothelial system (RES), the liver and spleen and negligible uptake by peripheral extremity skeletal muscles indicating limited utility of this approach for EVs delivery. Additional routes of administration to limb soft tissues such as intramuscular (IM) and subcutaneous (SC) injections, although guarantee direct EVs delivery to skeletal muscles, are localised to the site of injection and lack dose scalability due to the need for multiple doses and injection sites.

Transcatheter intravascular limb perfusion can present an efficient, clinically translatable approach for the targeted delivery of novel nanoscale therapeutics including EVs to limb skeletal muscles, vascularised composite tissue allografts (VCAs) or muscle flaps, both in situ and ex vivo. By exploiting the arterial bed of tissues for pharmaceutics administration, a given drug load can be delivered effectively and in a selective fashion. In fact, local extremity venous concentrations of a drug delivered via the IA route can reach values up to five times higher than concentrations that can be achieved using a systemic route of delivery for the same therapeutic agent (Avritscher and Javadi 2011; Stewart et al. 1983).

Additional advantages of IA perfusion‐based EVs delivery include homogenous and area‐wide distribution of the injectable substance to multiple skeletal muscle groups and simultaneous targeting of other extremity or free flap soft tissues including skin, nerves and vessels (Xu et al. 2018). IA perfusion can also circumvent the first pass effect, off‐set the non‐specific delivery of EVs and help optimise the homing of EVs to target skeletal muscles.

Here, we propose the pre‐reperfusion delivery of a pEVs‐enriched preparation via transcatheter IA regional limb infusion (RLI) for attenuating rhabdomyolysis and alleviating the concomitant cytokine storm following revascularisation of the ischaemic rodent extremity. First, we established and validated our novel catheter‐directed approach for therapeutic delivery to the rodent forelimb. Next, we characterised the safety and efficacy of transcatheter RLI for the administration of a cGMP‐grade pEVs‐enriched suspension in a rodent skeletal muscle IRI model. In addition, we investigated the potential therapeutic impact of delivering pEVs‐enriched preparations via this approach on alleviating the systemic sequel of skeletal muscle IRI.

## 2 Materials and Methods

### 2.1 Preparation and Characterisation of Clinical‐Grade Platelet‐Derived Extracellular Vesicles (pEVs) Formulation

Human pEVs‐enriched preparations utilised in these studies were obtained from Rion, Inc (Rion, Rochester, MN). Briefly, these formulations consist of concentrated EVs isolates derived from the conditioned medium of apheresis platelets sourced from human donors (Rolland et al. 2022). The conditioned medium is pooled before undergoing ultracentrifugation and staged filtration to eliminate all cytological debris and generate EVs, in a cGMP‐compliant fashion. pEVs from three different lots were characterised for size, morphology and contents as follows.

#### 2.1.1 Nanoparticle Tracking Analysis (NTA)

Particle size and concentration distribution of pEVs were measured using NTA (Malvern Instruments, Malvern, UK) according to the manufacturer's instructions. Briefly, pEVs samples were dissolved in 1X PBS and diluted to a final dilution of approximately 1:500–1:1000. Each sample analysis was conducted for 30 s and measured three times using NTA automatic analysis settings. The detection threshold was set to level 13 and camera level to 15.

#### 2.1.2 Transmission Electron Microscopy

Particle size and morphology were further assessed using transmission electron microscopy (TEM). Ten microlitres of the concentrated EV sample was deposited on a formvar/carbon‐coated copper grid and allowed to settle for 60 s. The sample was blotted and negative‐stained with a solution of 1% phosphotungstic acid (PTA) in water. The grids were blotted and allowed to air dry at room temperature. Grids were imaged with a JEOL JSM 1400 (JEOL, USA, Ltd, Peabody, MA) transmission electron microscope operating at 100 kV. Images were captured on a Veleta 2K × 2K CCD camera (Olympus‐SIS, Munich, Germany). Five to ten images were captured from each of three randomly selected areas of each grid with an acceleration voltage of 80 kV and an indicated magnification of 25,000X–80,000X. TEM micrographs were analysed manually. Spherical or ‘cup‐shaped’ particles with high‐contrast edges were considered EVs. EV diameters (average *N* = 100 vesicles per lot) were measured using Fiji plugin of ImageJ.

#### 2.1.3 Western Blotting

One vial of PEP (lot # 23001A) was used for the assay. To remove excess serum, the PEP cake powder was further purified using the ExoEasy Maxi Kit (Cat#: 76064; Qiagen, Hilden, Germany). Briefly, one vial of PEP was resuspended in 2 mL of sterile water and later filtered (cat# 16592; Minisart Syringe Filter, Surfactant‐free Cellulose Acetate Pore Size 0.8 µm; Gottingen, Germany). The remainder of the preparation was per Qiagen's manufacturer protocol.

The protein concentration was estimated using the BCA method (cat# 23225; Pierce, ThermoFisher, Waltham, MA), which resulted in a concentration of 1.17 µg/µL. Next, the sample was prepared for electrophoresis by loading 20 µg of protein and molecular marker (cat#: LC5925, SeeBlue Plus2 ladder, ThermoFischer, Waltham, MA) onto a separating gel (cat#: NW04122Box, Invitrogen Bolt 4%–12% Bis‐Tris Plus gel, ThermoFisher) in running buffer (cat#: NP0002, NuPage MES Running Buffer, ThermoFisher). This was transferred onto a PVDF membrane (cat#88520, ThermoFisher).

After blocking in 5% nonfat dry milk in 1X TBST for 1 h at room temperature, the membrane was later incubated with primary antibody overnight at 4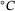. The antibodies used were anti‐Alix (ab275377; dilution of 1:5000, Abcam, Cambridge, UK), anti‐CD9 (ab236630; dilution of 1:1000, Abcam) and anti‐HSP 90 (ab53497; dilution of 1:2500, Abcam). The membrane was briefly washed in 1X TBST for 15 min and then incubated with the appropriate HRP linked secondary antibody (7076 and 7074; dilution of 1:5000; Cell Signaling Technology, Inc, Danvers, MA) for 1 h at room temperature. The membrane was washed in 1X TBST for 1.5 h. To visualise the proteins the enhanced chemiluminescent kit (cat #: A38556, Supersignal West Atto, ThermoFisher) was used for detection using the iBright CL1500 system (ThermoFisher). Following the detection of the target protein, the membrane was washed for 40 min in 1X TBST and blocked (5% nonfat dry milk in 1X TBST) to evaluate load control expression of beta actin (cat#: ab8227; dilution of 1:2800, Abcam).

#### 2.1.4 pEVs Protein Profiling

##### 2.1.4.1 EVs Lysates Preparation

Further characterisation of EV markers was performed using a bead‐based Luminex xMAP immunoassay (n = 4). Lyophilised pEVs powder was reconstituted in 2 mL of ice‐cold lysis buffer (Tris‐buffered saline, pH 7.4; 100 mM Tris, 150 mM NaCl; 1% NP‐40; 0.2% sodium deoxycholate; 1 mM EDTA; cOmplete Mini protease inhibitor cocktail, Roche). Samples were incubated on ice for ≥30 min with intermittent vortexing to lyse EV membranes, then centrifuged at 4000 × *g* for 15 min at 4°C. Supernatants were clarified through a low‐protein‐binding syringe filter before downstream testing. Total protein was quantified with the Pierce BCA assay using buffer‐matched standards/blanks. For the multiplex assays, lysates were adjusted to 5 mg/mL total protein and processed per the manufacturer's instructions; all samples were run in technical duplicates alongside a full standard curve.

##### 2.1.4.2 Human Exosome 9‐Plex Characterisation Panel

Nine markers were measured using Eve Technologies’ Human Exosome Characterization Panel 9‐Plex Featured Assay (HEX‐08‐08) based on the MILLIPLEX Human Exosome Characterization Magnetic Bead Panel (Cat. HEXSM‐170K, MilliporeSigma, Burlington, MA, USA). The 9‐plex comprised Argonaute‐2 (Ago‐2), calreticulin (CALR), CD9, CD63, CD81, Flotillin‐1 (FLOT‐1), GAPDH, Syntenin‐1 (Syn‐1) and TSG101. Ago‐2 is included by the manufacturer as a negative control for exosomes (‘exosome‐unenriched’ marker): exosome preparations typically yield background fluorescence intensity (FI/MFI), whereas cell lysates show a strong signal. Because no recombinant standard is provided for Ago‐2, results are reported as FI only and interpreted qualitatively with reference to the kit's positive control lysate. Results were normalised to protein content.

##### 2.1.4.3 TGF‐β 3‐Plex Discovery Assay Multi Species Array (TGF‐β1‐3)

TGF‐β1, TGF‐β2 and TGF‐β3 were quantified using Eve Technologies’ TGF‐β 3‐Plex Discovery Assay Multi‐Species Array per the manufacturer's instructions (MILLIPLEX TGFβ 1,2,3 Magnetic Bead Kit, Cat. TGFBMAG‐64K‐03, Millipore Sigma, Burlington, MA, USA). Results were normalised to protein content.

##### 2.1.4.4 Human Angiogenesis & Growth Factor 17‐Plex Discovery Assay Array (HDAGP17)

Seventeen analytes were measured using the Human Angiogenesis & Growth Factor 17‐Plex Discovery Assay (HDAGP17) (MILLIPLEX Human Angiogenesis/Growth Factor Magnetic Bead Panel 1, Cat. HAGP1MAG‐12K, Millipore Sigma) per the manufacturer's instructions. The panel included Angiopoietin‐2, BMP‐9, EGF, Endoglin, Endothelin‐1, FGF‐1, FGF‐2, Follistatin, G‐CSF/CSF‐3, HB‐EGF, HGF, IL‐8/CXCL8, Leptin, PlGF, VEGF‐A, VEGF‐C and VEGF‐D. Results were normalised to protein content. Multiplexing was performed by Eve Technologies (Calgary, Alberta, Canada) on a Luminex 200 system (Luminex/DiaSorin) with Bio‐Plex Manager software (Bio‐Rad). Analytes were normalised to protein content.

#### 2.1.5 Small RNA Sequencing and miRNA Profiling

Three distinct vials of pEVs from separate lots were reconstituted in 1 mL of TRIZol each followed by chloroform extraction as per standard RNA extraction protocols. Quality of extracted RNA was assessed using an RNA Integrity Number (RIN) and a DV200 value generated by the Agilent Fragment Analyzer (Agilent, Santa Clara, CA). Qualitative concentration is determined using a Qubit Fluorometer (ThermoFisher Scientific, Waltham, MA).

Small RNA libraries were prepared from 1 ng of total RNA according to manufacturer's guidelines for QIAGEN's QIAseq miRNA Library Kit (QIAGEN, Germantown, MD). Briefly, adaptors were ligated to the 3′ and 5′ ends of the miRNAs as part of total RNA samples. A complementary primer was annealed to 3′ adaptor sequences followed by reverse transcription to generate a cDNA library of small RNAs. cDNA libraries were purified followed by an enrichment step in which a unique index was added to each sample. A final purification step was performed prior to quantitation of completed libraries using Agilent Bioanalyzer and Qubit fluorometer.

Libraries were sequenced at an average coverage of ∼75 M read pairs following Illumina's standard protocol for Illumina NextSeq2000 P1 flow cell. The flow cells will be sequenced as 100×1 single end reads using NextSeq2000 P1 sequencing kit and NextSeq Control Software v1.5.0. Base‐calling is performed using Illumina's RTA version 3.4.4.

Secondary computational analysis of raw microRNA sequencing data was processed using the Qiagen workflow available on GeneGloble Data Analysis Center. Briefly, FASTQ files were uploaded and underwent adapter trimming, quality filtering and alignment to the miRBase database and reference genome to quantify miRNA expression. Tertiary analysis was performed in R (v.4.4.1) for. Raw counts were normalised for sequencing depth using the TMM Normalisation process (trimmed mean of M component).

### 2.2 Animals and Forelimb Ischaemia‐Reperfusion Injury Model

All experimental procedures adhered to guidelines approved by the Mayo Clinic Institutional Animal Care and Use Committee (IACUC) (A00005926‐21) and complied with the ARRIVE (Animal Research: Reporting of In Vivo Experiments) guidelines. Thirty healthy adult male (*n* = 19) and female (*n* = 11) Sprague‐Dawley (SD) rats (Envigo, IN) weighing 300–450 g were used for both the pilot in vivo study (*n* = 25) and biodistribution study (*n* = 5). Animals were allowed to acclimate for 5 days before their use in experimentation. Rats were housed in groups of two to three animals per plastic cage before surgery and caged separately postoperatively. They were kept in standard conditions – 12 h/12 h light/dark cycle, temperature 22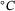 and unrestricted access to commercial rodent chow and water ad libitum. All live surgical procedures were conducted under isoflurane inhalation anaesthesia: 3%–4% vapourised isoflurane for the induction of anaesthesia and 1.5%–2% for maintenance anaesthesia. Animals were placed in supine position over a heating pad maintained at 37°C to prevent hypothermia during surgery. Subcutaneous Buprenorphine (1 mg/mL) was given immediately before the surgical procedure and tourniquet placement for pain control. To prevent dehydration, 10 mL of sterile normal saline (0.9% sodium chloride) was administered subcutaneously during the perioperative period. Animals were monitored for signs of pain, suffer or distress postoperatively until sacrifice.

To simulate ischaemic/reperfusion conditions of the peripheral extremity as in acute limb ischaemia, major limb replantation and/or upper extremity transplantation, we used a tourniquet rat forelimb IRI model (Figure 3A). We have previously demonstrated that our tourniquet‐induced forelimb ischaemia model induces severe metabolic derangements, immune dysfunction and massive limb swelling, recapitulating the hallmarks of extremity post‐reperfusion syndrome (Selim et al. 2025). Details of tourniquet placement and forelimb blood flow evaluation are described in Sections 2.3.1 and 2.6.

### 2.3 Groups

To determine the safety of RLI and potential myoprotective of pEVs on attenuating skeletal muscle IRI and stabilising the myocyte membrane, we devised a pilot in vivo study comparing animals receiving pEVs via a regional IA delivery method to animals given 50 mM trehalose, a commonly used lyoprotectant. Trehalose is an anti‐caking or exosome excipient agent that reduces EVs aggregation and preserves EVs membrane integrity during lyophilisation process, a process also known as lyoprotection (Golan and Stice 2024; El Baradie et al. 2020). Thus, agents like trehalose might help enhance the integrity and stability of EVs required in standardised large‐scale production of pharmaceutical‐grade EVs. A total of 25 SD rats (14 males and 11 females) was used for this pilot in vivo study.

#### 2.3.1 Group A (Untreated Controls/Wild‐Type ‘WT’)

This group consists of WT SD rats that did not undergo any surgical procedure or treatment; however, they were used to provide baseline normal values of serum clinical chemistry and cytokines (*n* = 10; 6 males and 4 females). Three male rats were used as control for Xenogen IVIS experiments and ex vivo biodistribution studies.

#### 2.3.2 Group B (IRI/Saline–Trehalose)

This group underwent tourniquet‐induced forelimb IRI with tourniquet ischaemia time of 150 min and received 500 µL of 50 mM trehalose (Trehalose dihydrate, 625625, Sigma–Aldrich, MO) in 50 U/mL heparinised normal saline via locoregional IA limb perfusion 30 min before tourniquet release. Animals were then followed up for 24 h and sacrificed for preliminary biochemical analysis (*n* = 8; 4 males and 4 females).

#### 2.3.3 Group C (IRI/Saline‐pEVs)

This group underwent tourniquet‐induced forelimb IRI with tourniquet ischaemia of 150 min and received 500 µL of approximately 2×10^11^ particles/mL in 50 U/mL heparinised normal saline via locoregional IA limb perfusion 30–45 min before tourniquet release. Dose optimisation was not performed, and dosage selection was based on prior on previous EV studies using a similar dosage of this pEVs formulation. Animals were then followed up for 24 h and sacrificed for preliminary biochemical analysis (*n* = 7; 4 males and 3 females).

### 2.4 Microsurgical Approach for Transcatheter Intra‐Arterial Delivery to the Vascularly Isolated Rat Forelimb

Regional IA limb infusion (RLI) of therapeutic EVs or control treatment was performed via a selective brachial artery microcatheterisation approach. A surgical dissecting microscope (Zeiss, Germany) was used to perform brachial artery dissection and cannulation under high magnification. All surgical procedures were performed by a single investigator (OAS) to ensure consistency and replicability. Because the pEVs‐enriched suspension is visibly translucent yellow upon reconstitution, whereas the vehicle is transparent, blinding of the injector was not feasible; however, downstream assays were performed under investigator‐blinded conditions and animals were assigned by adaptive randomisation to reduce procedural bias.

#### 2.4.1 Induction of Forelimb Ischaemia and Temporary Occlusion of Forelimb Collateral Circulation

Following isoflurane induction and animal preparation, a cohesive band was wrapped around the rat forelimb starting at the hand distally and ending at the shoulder joint proximally to exsanguinate the forelimb before tourniquet application. Next, a silicone digital exsanguinating tourniquet (Tourni‐Cot, Mar‐Med) was placed at the level of the shoulder joint to temporarily isolate the forelimb circulation and induce acute peripheral limb ischaemia for 150 min. Tissue ischaemia was confirmed by LSCI, absence of brachial artery pulsations using a digital doppler and inspecting for forepaw skin pallor.

#### 2.4.2 Surgical Exposure

A 10 mm skin incision was placed along the longitudinal axis of the rate brachium proximal to the antecubital fossa to expose the forelimb neurovascular pedicle (Figure 2D, i). The underlying SC tissue was dissected bluntly, and a self‐retaining retractor was used to facilitate the exposure of deeper soft tissues including the brachium major blood vessels.

#### 2.4.3 Blunt Dissection of the Rat Forelimb Neurovascular Pedicle

Next, the basilic and cephalic veins and brachial artery were meticulously dissected using sterile Q‐tips and fine‐tipped microsurgical forceps. A background material was then placed posterior to the brachial artery to separate the artery from surrounding structures, facilitate microcatheter introduction and provide tissue support and contrast (Figure 2D, ii). The field was constantly irrigated with 2% lidocaine to prevent vasospasm.

#### 2.4.4 Brachial Arteriotomy and Microcatheterisation

Vascular access to the forelimb arterial supply was achieved by carefully creating an arteriotomy (incision in arterial wall) in the anterior wall of the brachial artery using the bevelled tip of a 32G needle held by a Castroviejo microneedle holder (Figure 2D, iii and iv). Next, a specialised vessel cannulation forceps (Roboz Surgical Instruments Co., Gaithersburg, MD) was used to introduce a polyimide microcatheter connected to a four‐way stopcock (Doccol Corporation, Sharon, MA) with an outer diameter: 0.191 mm and inner diameter: 0.152 mm (Figure 2D, v). Slipknots using 7‐0 silk suture were applied over the distal portion of the microcatheter to prevent backflow of injectable substance and secure the microcatheter in place (Figure 2D, vi). Low‐pressure infusion of 500 µL of the injectable substance such as pEVs was then infused over a period of 30 min.

#### 2.4.5 Repair of Brachial Arteriotomy, Haemostasis and Skin Closure

At the end of the infusion, the catheter was withdrawn gently, and the arteriotomy was repaired using a 9‐0 nylon simple suture (Figure 2D, vii and viii). A fat pad was placed around the vessel and inserted before tying the final knot. Skin closure was performed using a 5‐0 Vicryl running suture (Ethicon). At the end of the ischaemia period, the tourniquet was released, and haemostatic (occlusive) pressure was applied at the surgical site for 5–10 min, and restoration of forelimb blood flow was confirmed by forepaw skin colour change, digital doppler assessment and laser speckle contrast imaging (LSCI, please see below for method details).

### 2.5 Validation of Regional Intra‐Arterial Limb Infusion (RLI) Technique for Therapeutic Delivery

Preliminary validation of our microsurgical technique was undertaken in both cadavers and live rats (*n* = 3–4) via biplane fluoroscopy and in vivo dye injection. In cadaver testing, normal WT SD rats were euthanised by terminal exsanguination to remove blood from the peripheral vasculature to facilitate contrast injection before use in forelimb angiography studies. For the in vivo validation study, animals were anaesthetised similarly as described above, and 1% Evans blue dye (EBD) was injected as a surrogate marker of any biologic therapeutic directly into the brachial artery. At the end of the infusion, the biodistribution of the dye was determined by examining the upper extremity soft tissues (skeletal muscles, nerves and skin) for any bluish discolouration.

To confirm the placement and position of the microcatheter, we performed biplane fluoroscopy and digital subtraction angiography (DSA) on three rats. Briefly, 500 µL of iohexol (Omnipaque 350 GE Healthcare, Marlborough, MA) were infused using our RLI protocol. The specimens were then imaged on a biplane fluoroscopy angiography system (Artis zee biplane, Siemens Healthineers GmbH). The acquisitions were performed with a DSA protocol using a tube potential of 63.8 kV, tube current of 422 mA and a frame rate of 2 frames per second. The imaging field of view was set to 80 mm, and the resulting images had a 512 × 512‐pixel matrix.

### 2.6 Blood Flow Studies

We used both qualitative and quantitative methods to evaluate the safety of the transcatheter IA approach for therapeutic delivery to limbs, and hence the safety of IA pEVs delivery to limb skeletal muscles. In addition, our forelimb ischaemia and reperfusion model were confirmed using the following.

#### 2.6.1 Full‐Field Laser Speckle Contrast Imaging (LSCI)

LSCI is a non‐invasive and non‐contact optical imaging modality that detects and quantitively evaluates areas of dynamic microcirculatory perfusion without the need of contrast dye injection or prohibitive imaging apparatus. The basic principle of LSCI relies on motion‐induced turbulence of speckle patterns arising from the backscattering of coherent laser light by a scattering medium such as biological tissues. Moving objects such as RBCs flowing inside vessels for instance can thus result in a dynamic change in speckle patterns that can be detected by a global shutter camera with complementary metal‐oxide semiconductor (CMOS) sensors. As these dynamic speckles contain information about change in particles or RBCs motion over time, special analytical approaches and formulas can be utilised to extract information about blood flow in the scanned tissue.

In our study, a commercially available LSCI platform (RFLSI‐ZW, RWD Life Science, Guangdong, China) was utilised for quantifying cutaneous microcirculatory blood flow in our rodent model as an indirect measure of the upper extremity perfusion. The region‐of‐interest (ROI) analysed for blood flow assessment was anatomically defined by the angiosome provided by the rat brachial artery and its two major branches; the median and ulnar arteries, whose distribution spans the antecubital fossa proximally and the fingertips distally.

On average, the rat forelimb was scanned for 30 s, and a time‐of‐interest (TOI) of at least 15 s was selected from real‐time graphs to exclude motion‐induced artefacts and reduce temporal variability of perfusion measurements (Figure 2). The following scanning parameters were used to acquire LSCI flux perfusion data: exposure time: 20 ms, filter constant: 3 s, frame rate: 50 fps, camera gain: ∼152 and algorithmic mode: spatial algorithm (sliding mode). Mean and standard deviation of perfusion units [arbitrary units (a.u.)] were calculated from the selected TOI flux data generated using the manufacturer image analysis software. The mean perfusion unit for each animal was then used in downstream statistical analysis.

#### 2.6.2 Brachial Artery Doppler Assessment and Waveform Analysis

A handheld digital Doppler (Dopplex DMX Digital Doppler, Huntleigh, UK) was used to qualitatively validate ischaemia and reperfusion in our model by assessing brachial artery flow under the different conditions. Brachial artery Doppler tracings were exported and analysed using Dopplex Vascular Reporter (Huntleigh, Arjo, UK). Tracings were interpreted for the absence or presence of normal arterial triphasic pulsatile waveform components.

### 2.7 Blood and Skeletal Muscle Tissue Harvest

At the end of the experiment, animals were euthanised humanely via exsanguination. Terminal blood collection was obtained by a cardiac puncture for serum analysis (Figure 7). At least 4–8 mL of blood was obtained by cardiac puncture. Whole blood was allowed to clot at room temperature for at least 1 h before serum was isolated by centrifugation at 4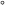 C for 15 min at a speed of 1800 × *g*. The serum samples were aliquoted in 200 µL and stored in sterile plastic tubes at −80°C until use for serum clinical chemistry and cytokine profiling.

Skeletal muscles of the forelimb [flexor carpi ulnaris (FCU), FCR, PL, flexor digitorum profundus (FDP), FDS, and extensor carpi radialis longus and brevis] were immediately dissected and harvested. Muscles were snap‐frozen in isopentane prechilled in liquid nitrogen and stored at −80°C until further analysis.

### 2.8 Ex Vivo Biodistribution Study Following RLI Delivery (*n* = 8)

#### 2.8.1 EVs Fluorescent Labelling

To characterise pEVs localisation in skeletal muscle tissue, EVs were first labelled by membrane integration of the lipophilic dye 1,1′‐dioctadecyl‐3,3,3′,3′‐tetramethylindocarbocyanine perchlorate (‘DiI’ D3911, ThermoFisher). Briefly, pEVs powder cake was dissolved in 1X PBS buffer, and DiI in anhydrous DMSO was transferred to achieve a final concentration of 20 µM. The labelling was completed at 37°C under agitation for 30 min. Next, the unreacted dye was separated from the DiI‐labelled pEVs suspension by using Amicon protein sample ultrafiltration (50 kDa molecular weight cutoff, Millipore). Labelled pEVs were washed thrice using 1X PBS.

Five male SD rats were used for pEVs biodistribution study. DiI‐labelled EVs were administered using the same transcatheter IA limb delivery approach (Figure 4A). Following a 24‐h reperfusion period, the rats were euthanised humanely, and forelimb skeletal muscles and systemic organs were harvested for fluorescent pEVs quantification and localisation.

#### 2.8.2 Gross Visualisation of pEVs Biodistribution in Tissues Using IVIS Imaging

Fluorescent images of skeletal muscle tissues and organs were acquired using the in vivo imaging system (Xenogen IVIS; PerkinElmer) (Figure 6A). Image scans were acquired at an excitation wavelength of 535 nm and an emission wavelength of 580 nm. Ex vivo organ epifluorescence signal was analysed and quantified using the software program Living Image (PerkinElmer). Results were expressed as total radiant efficiency in units of photons/second within the region of interest (ROI) $\frac{p/s/cm^{2}/\mathrm{sr}}{\mu W/cm^{2}}$.

#### 2.8.3 Microscopic Visualisation of pEVs Biodistribution in Tissues Using Confocal Microscopy

Following IVIS imaging, FDP muscles from both forelimbs, the major extrinsic wrist flexor, were embedded in Optimal Cutting Temperature (OCT) compound (Sakura, Tissue‐Tek) and snap frozen using an isopentane bath prechilled in liquid nitrogen. Next, 7‐µm thick frozen sections were cut using a standard cryostat (Leica CM1850, Leica Biosystems, IL) and loaded onto glass microscope slides (Fisherbrand Superfrost Plus Microscope Slides, Fisher Scientific, MA).

Immunohistochemical staining for laminin was then performed to visualise skeletal myofibre sarcolemma. Briefly, frozen sections were fixed with 4% paraformaldehyde (Electron Microscopy Services) for 15 min and then washed in 0.1 M glycine for quenching unreacted aldehydes. Fixed skeletal muscle sections were then permeabilised with 1% Triton‐X for 30 min and then blocked using 5% foetal bovine serum/bovine serum album in 1X PBS for 45 min. Frozen sections were then incubated with anti‐laminin (D18) antibody, 1:100 dilution (Developmental Studies Hybridoma Bank) for overnight at 4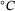. Primary antibody was removed by washing slides with 0.1% triton‐X in 1X PBS for 15 min thrice. Fluorescent detection of laminin was done by staining slides using Alexa Fluor 488 goat anti‐mouse (A‐11001; 1:500; ThermoFisher). All sections were mounted in Fluoroshield with DAPI (ab104139, Abcam) and visualised using a high‐resolution laser scanning confocal microscope (Zeiss LSM980, Germany)

### 2.9 Preparation of Skeletal Muscle Tissue Homogenates

Snap‐frozen flexor (FCU, FCR, FDS and PL) and extensor (ECRB and ECRL) antebrachium muscles from the operative and contralateral (non‐operative) forelimbs were cryopulverised under liquid nitrogen with a pre‐chilled mortar and pestle to a fine powder. Tissue powder was resuspended in at least 5 mL of ice‐cold lysis buffer (1× PBS, 1% NP‐40, 1 mM EDTA, cOmplete Mini protease inhibitor cocktail, Roche), incubated on ice for ≥60 min with intermittent vortexing and centrifuged at 4000 × *g* for 10 min at 4°C. The supernatant was clarified through a low‐protein‐binding syringe filter and used for downstream assays. The total protein concentration of tissue homogenates was determined using the Pierce BCA assay. Homogenates were then aliquoted into 2‐mL sterile PCR tubes, snap‐frozen in liquid nitrogen and stored at −80°C until analysis, avoiding repeated freeze–thaw cycles.

### 2.10 Serum Chemistry

Clinical biomarkers of skeletal muscle and kidney injury circulating in serum were analysed: creatine kinase (CK, U/L), aspartate transaminase (AST, U/L), lactate dehydrogenase (LDH, U/L), alanine transaminase (ALT, U/L), creatinine (Cr., U/L), blood urea nitrogen (BUN, mg/dL), potassium (K^+^, mEq/L) and sodium (Na^+^, mEq/L) levels were quantified spectrophotometrically using a clinical chemistry analyser (IDEXX Bioanalytics, Grafton, MA).

### 2.11 Serum and Skeletal Muscle Tissue Cytokine Profiling

To evaluate the anti‐inflammatory effect of pEVs, we used Luminex xMAP technology for multiplex cytokine profiling of serum and forelimb skeletal muscle tissue samples at 24 h post‐reperfusion. A Rat Cytokine/Chemokine 27‐Plex Discovery Assay Array (RD27) as per the manufacturer's instructions for use (MILLIPLEX Rat Cytokine/Chemokine Magnetic Bead Panel Cat. # RECYTMAG‐65K, MilliporeSigma, Burlington). The 27‐plex consisted of EGF, Eotaxin/CCL11, Fractalkine/CX3CL1, G‐CSF/CSF‐3, GM‐CSF, GROα/CXCL1/KC/CINC‐1, GROβ/CXCL2/MIP‐2/CINC‐3, IFN‐γ, IL‐1α, IL‐1β, IL‐2, IL‐4, IL‐5, IL‐6, IL‐10, IL‐12(p70), IL‐13, IL‐17A/CTLA‐8, IL‐18, IP‐10/CXCL10, Leptin, LIX, MCP‐1/CCL2, MIP‐1α/CCL3, RANTES/CCL5, TNF‐α and VEGF‐A. The multiplex analysis was tested using the Luminex 200 system with Bio‐Plex Manager software (Eve Technologies, Calgary, Alberta, Canada). Samples associated with aggregated, low bead counts were eliminated according to the manufacturer (serum, *n* = 1).

### 2.12 Skeletal Muscle Tissue Homogenates Transforming Growth Factor‐β Levels

Undiluted skeletal muscle tissue lysates were analysed for TGF‐β1, TGF‐β2 and TGF‐β3 using a bead‐based 3‐plex immunoassay (Eve Technologies, TGFB 3‐Plex Discovery Assay Multi Species Array) based on the MILLIPLEX TGFβ 1,2,3 Magnetic Bead Kit (Cat. TGFBMAG‐64K‐03; MilliporeSigma, Burlington), following the manufacturer's instructions. Assay calibration was performed with kit‐provided recombinant standards, and concentrations were reported in pg/mL (Eve Technologies Corporation, Calgary, Canada). Samples and standards were run in technical duplicates, and standard curves were fit with a five‐parameter logistic (5‐PL) model on the Luminex xMAP platform. Homogenate cytokine concentrations were normalised to total protein in the lysate and reported as pg/mg protein.

### 2.13 Enzyme Linked Immunosorbent Assay (ELISA) for Determination of Protein Carbonyls

Protein carbonyls, a stable, irreversible marker of oxidative protein damage, were quantified in serum and skeletal‐muscle homogenates using a commercial ELISA kit based on 2,4‐dinitrophenylhydrazine (DNPH) derivatisation, followed by anti‐DNP antibody detection, per the manufacturer's instructions (OxiSelect Protein Carbonyl, STA‐310). Total protein in each sample was first determined by BCA, and samples were adjusted to the kit's recommended working concentration of 10 µg/mL. Serum was initially diluted 1:200 in 1× PBS and then further adjusted as needed to achieve 10 µg/mL; tissue homogenates were similarly normalised by protein. Standards and samples were run in technical duplicates and fitted with a four‐parameter logistic (4PL) regression to interpolate unknowns from the standard curve. Samples that fell below the limit of detection (LOD) of the standard curve were not included in the analysis. Results were blank corrected where applicable and expressed as nmol carbonyl per mg total protein (nmol/mg).

### 2.14 Skeletal Muscle Histology and Semi‐Quantitative Analysis

The FDP muscle, major extrinsic wrist flexor, was fixed in formalin for at least 48 h at room temperature before embedding in paraffin. Five microns thick sections were obtained and stained using haematoxylin and eosin Y according to standard protocols. A validated histological scoring system with modification was used to evaluate histologic scoring (Brouwers et al. 2022). Three‐parameter scoring was based on evaluating three processes: (1) inflammation, (2) myofibre degeneration and damage and (3) variation in shape and size of myocytes.

### 2.15 Statistical Analysis

All data are presented as the mean ± standard error of mean unless otherwise stated. All statistical analyses were performed using GraphPad Prism version 10.4.1, and *P* values < 0.05 was considered statistically significant. Comparisons between multiple groups were compared using a one‐way analysis of variance (ANOVA) with Tukey's post hoc test. Skeletal muscle tissue and organ biodistribution data analysis was performed using two‐way ANOVA followed by Bonferroni post‐hoc analysis. Outliers were detected using ROUT method, robust nonlinear regression and outlier removal, using a *Q* = 2% (Motulsky and Brown 2006). For Luminex immunoassays, undetectable analyte values below the assay's lower LOD were replaced with LOD/2 before analysis. No power analysis was performed for this study.

## 3 Results

### 3.1 Characterisation of cGMP‐Compliant, Lyophilised Platelet‐Derived EVs‐Enriched Formulation

**FIGURE 1:**
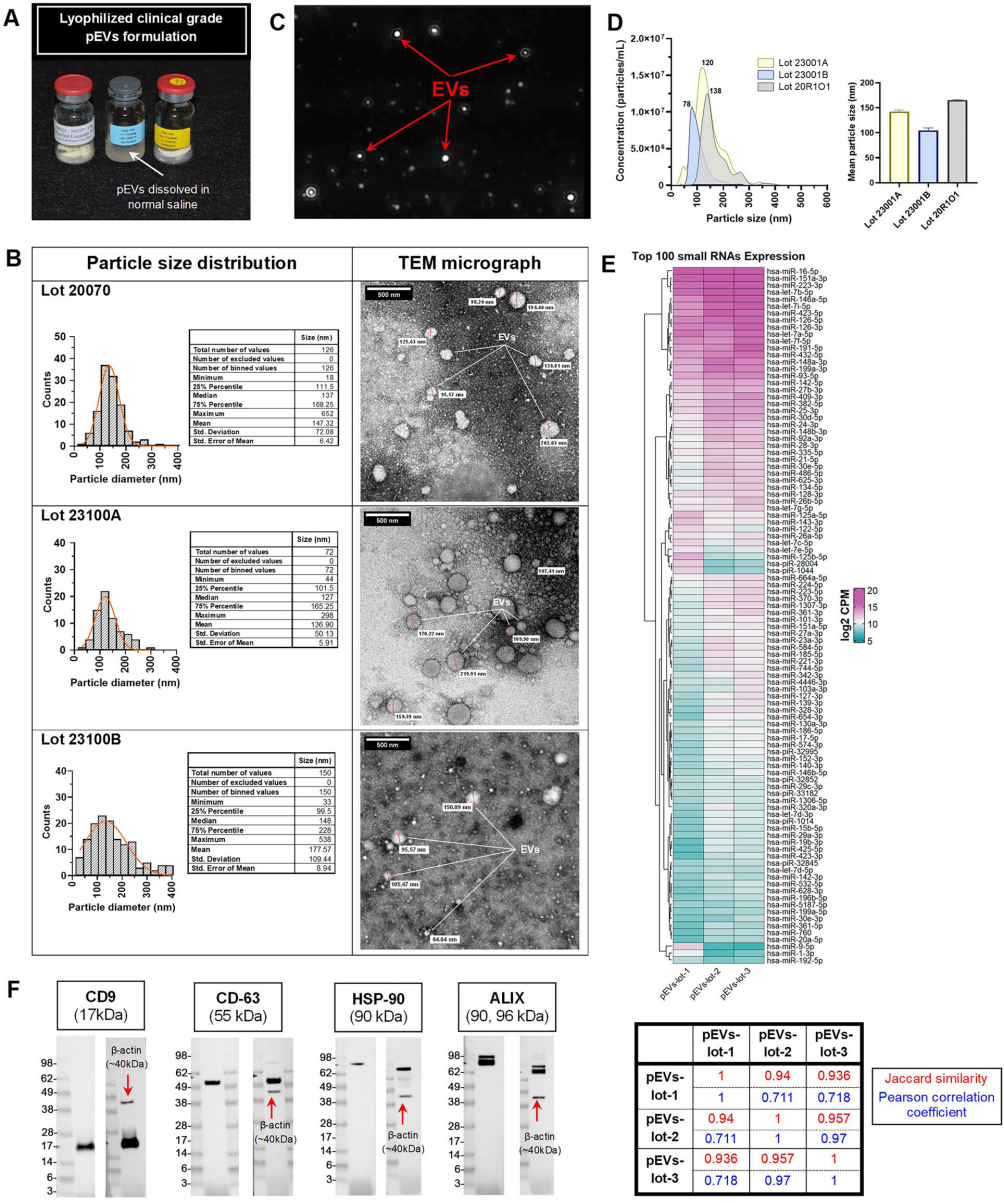
Characterisation of an off‐the‐shelf, lyophilised platelet‐derived EVs (pEVs)‐enriched preparation: (A) Lyophilised clinical‐grade pEVs is supplied as a ‘ready‐to‐use’ powder cake (75 mg) in sterile glass vials. The pEVs powder cake is soluble in aqueous media such as normal saline, and its dissolution yields a yellowish, translucent solution (middle vial). (B) Representative snapshot video from the Nanosight instrument showing light scatter from platelet‐derived particles on NTA. (C) Particle size distribution and concentration measured by nanoparticle tracking analysis. (D) EVs morphology and particle size distribution of three different lots measured by TEM; (E) Top 100 small RNAs expression across pEV lots. Heatmap shows log_2_‐CPM values (TMM‐normalised) for the 100 most abundant small RNAs across pEV‐lot1, pEV‐lot2 and pEV‐lot3. Rows and columns were hierarchically clustered (Euclidean distance, complete linkage). Colour scale indicates relative abundance (cyan = low, magenta = high). Top: EV miRNA signature for three lots (top‐100 enriched species). Bottom: Pairwise similarity between lots: Jaccard index for top‐100 membership (lot‐1/lot‐2 = 0.94; lot‐1/lot‐3 = 0.936; lot‐2/lot‐3 = 0.957) and Pearson correlation of log_2_‐CPM abundance (0.711, 0.718 and 0.97, respectively). (F) Western blot analysis of a representative lot (23100A) revealed enriched EVs‐specific markers CD63, CD9 and ALIX. Dual probe blot was performed (right) for each representative blot with loading control (β‐actin). Vials of this lot were used for downstream in vivo testing. EV, extracellular vesicle; NTA, Nanoparticle Tracking Analysis; TEM, transmission electron microscopy.

Human pEVs were characterised for morphology, particle size and surface markers (Figure 1A). TEM showed particles exhibiting cup‐shaped or spherical morphology typical of EVs following negative staining using 1% PTA (Figure 1B). Further TEM image analysis revealed mean particle size of 136.90 ± 5.91, 147.32 ± 6.42 and 177.57± 8.94 nm for the three different lots of pEVs‐enriched preparation. TEM results were further corroborated using NTA that showed a comparable particle size distribution and mean diameter for the three different lots tested (Figure 1C, D). Furthermore, the mean concentration of the three different lots ranged from 6.05$e^{8}$ to 1.31$e^{9}$ particles/mL with a combined mean of 9.52$\;e^{8}$ particles/mL.

The heatmap of the top 100 most abundant small RNAs (log_2_‐CPM, TMM‐normalised) shows that all three lots share a common miRNA signature (top panel, Figure 1E). Set overlap of the top‐100 lists was high: Jaccard indices were 0.94 for lot‐1 versus lot‐2, 0.936 for lot‐1 versus lot‐3 and 0.957 for lot‐2 versus lot‐3 (bottom panel, Figure 1E), indicating >93% concordance in miRNA identity across lots. Pairwise Pearson correlations of miRNA abundance (log_2_‐CPM) showed very high concordance between lot‐2 and lot‐3 (*r* = 0.97), with moderate concordance for lot‐1 versus lot‐2 (*r* = 0.711) and lot‐1 versus lot‐3 (*r* = 0.718). Thus, while the composition of highly expressed miRNAs is largely shared, lot‐1 exhibits modest abundance shifts relative to the other two lots. Because each lot was profiled once, we did not perform inferential testing across lots. Instead, we quantify reproducibility using set overlap (Jaccard) and abundance correlation (Pearson), which together support a stable core miRNA cargo with minor inter‐lot variation in relative levels. In accordance with the *Minimum Information for Studies of Extracellular Vesicles* 2023 (MISEV2023) guidelines, western blot analysis of samples from the lots used in this study demonstrated enrichment of several characteristic EVs biomarkers including tetraspanins such as CD9 and CD63, chaperones (heat shock proteins) such as HSP90, and EVs biogenesis proteins such as ALIX (Figure 1F). Multiplexed protein profiling of EV lysates confirmed EV identity across vials from representative lots (Table S1). MISEV2023‐guided protein analysis showed positive canonical transmembrane markers, CD63 and CD9 (MISEV2023, Category 1/2) and cytosolic TSG101 (MISEV2023, Category 2a). Non‐EV‐specific proteins, CALR (MISEV2023, Category 4c) and GAPDH (MISEV2023, Category 2b) were also measurable, indicating partial co‐isolation of endoplasmic reticulum (ER)/cytosolic material.

Moreover, the pEVs used in this study demonstrated the presence of proangiogenic mediators such as angiopeotin‐2, endothelin‐1, endoglin, fibroblast growth factor‐2 and vascular endothelial growth factor‐A and C were also abundant across the different preps (Table S1). Furthermore, multiplexed immunoassays detected comparable levels of TGF β1 and 2. Across four vials, approximately 50% of analytes showed CV ≤15% (good lot agreement), with higher variability for some EV/purity markers (CD9, TSG101 and CALR) and low‐abundance growth factors denoting a modest degree of lot‐to‐lot variability.

### 3.2 Validation of Catheter‐Directed Regional Limb Infusion (RLI) Technique for Therapeutic Delivery

**FIGURE 2:**
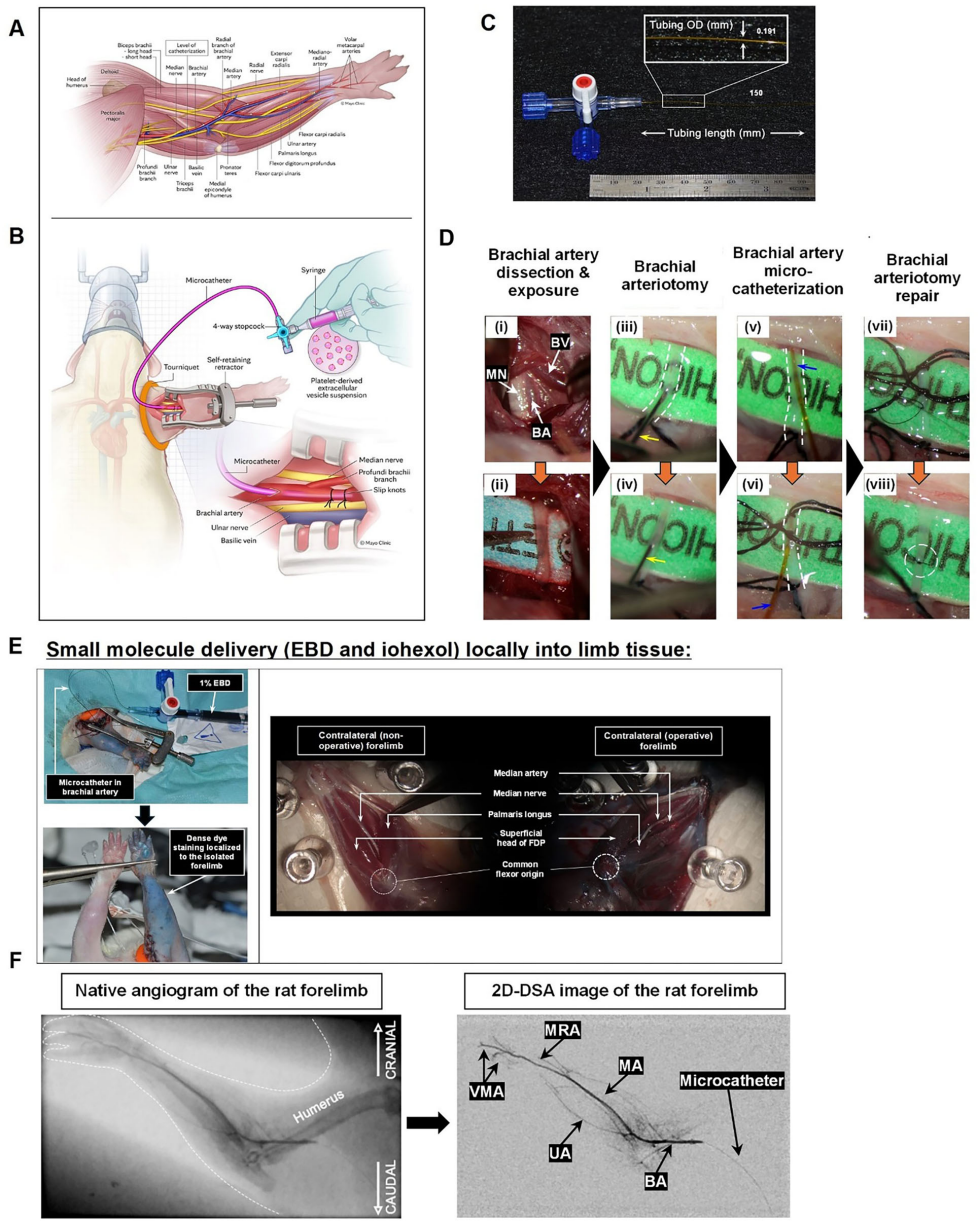
Transcatheter intra‐arterial regional limb infusion (RLI) strategy for delivery of therapeutics to the peripheral extremity: (A) Pertinent surgical anatomy of the rat forelimb. Level of brachial artery catheterisation is indicated. (B) Schematic outlining the set‐up of selective brachial artery catheterisation for local platelet‐derived extracellular vesicles (pEVs) delivery to the vascularly isolated rodent extremity. (C) Polyimide microcatheter used in this study for small vessel catheterisation with dimensions; OD: outer diameter. (D) Representative intraoperative photomicrographs of the critical steps of the surgical cutdown and brachial artery cannulation procedure using a dissecting microscope (magnification 10‐15X). (E) Acute experiments for validation of regional limb infusion (RLI) technique for small molecule delivery using Evans blue dye (EBD) and iodine‐based contrast medium. Dense dye staining localised to the area of isolated forelimb while the contralateral (non‐operative) side was spared. Bluish discolouration of the muscle and skin tissues. (F) Representative biplane fluoroscopy and digital subtraction angiography (DSA) of the rat forelimb immediately following Omnipaque 350 infusion using our established technique. DSA via brachial artery microcatheterisation (right panel) confirms catheter position. Rat forelimb arteries and catheter are depicted on the angiograms.

To target the rat forelimb, our approach to drug delivery was via brachial artery microcatheterisation proximal to the elbow joint (Figure 2A, B). We performed acute experiments using iodinated radiographic contrast medium and azo dye to qualitatively validate our technique and confirm catheter tip position (Figure 2E, F). Selective brachial artery DSA with IA administration of 500 µL of iohexol was technically feasible and clearly localised the position of our endovascular microcatheter (Figure 2F; right panel). Native angiograms revealed the placement of the microcatheter approximately 1 cm proximal to the antecubital fossa at the midhumeral level and antegrade flow following contrast infusion. Complete visualisation of the main trunk of the brachial artery and major distal branches (MA: median artery, UA: ulnar artery, MRA: mediano‐radial artery and VMR: volar metacarpal arteries) can also be appreciated from the 2D DSA image sequences indicating the locoregional selectivity of our approach (Figure 2F and Figure S1).

To further validate DSA findings from the macroscopic standpoint, we also injected EBD directly to the rat forelimb using our transcatheter approach. EBD is a non‐toxic, vascular impermeable dye that has affinity for albumin, making it a reliable tracer for macromolecule extravasation. Gross examination of the forelimb soft tissues revealed discolouration of the soft tissues, chiefly skeletal muscles and skin, of the operative, ischaemic forelimb while the contralateral (non‐operative) forelimb and rat corpus were spared further validating the ability of our RLI technique to selectively target the limb vasculature (Figure 2F).

### 3.3 Confirmation of Tourniquet‐Induced Forelimb Ischaemia‐Reperfusion Injury Model and Assessment of Safety of pEVs Delivery Following Delivery via Catheter‐Directed Regional IA Limb Infusion (RLI)

**FIGURE 3:**
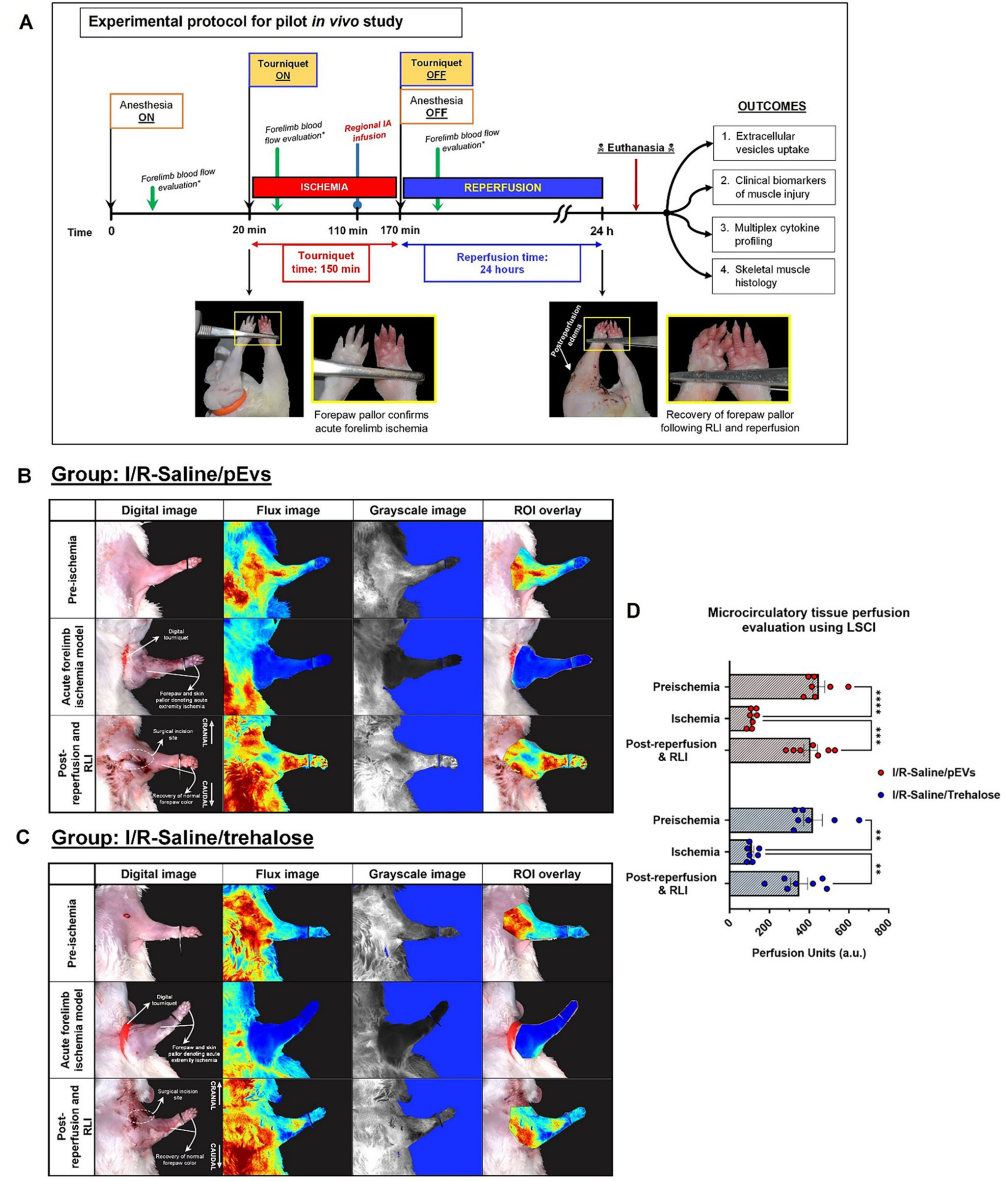
Experimental protocol for catheter‐directed IA RLI delivery in a forelimb IRI model and perfusion assessment for experimental groups. Forelimb perfusion measurements for I/R groups receiving saline‐pEVs or saline‐trehalose. (A) Schema and timeline for combined forelimb ischaemia‐reperfusion injury (I/R) and RLI experimental protocol. Insets depict monitoring forepaw skin colour for confirmation of acute limb ischaemia and recovery of forelimb blood flow following intra‐arterial delivery and tourniquet release. At 24 h, massive post‐reperfusion limb oedema occurs following tourniquet release (left) and surgical wound site can be seen healed in the representative photograph. (B) Representative laser speckle contrast imaging (LSCI) scans of operative forelimbs of rats receiving saline‐pEVs under different conditions. (C) Representative LSCI scans of I/R‐saline/trehalose group. (D) Perfusion quantification using LSCI for both groups under different conditions (*n* = 7 per group). Data presented as mean ± SEM. Statistical comparisons were performed using two‐way ANOVA with Tukey's post hoc analysis. **p < 0.01, ***p < 0.001. ANOVA, analysis of variance; EV, extracellular vesicle; IA, intra‐arterial; IRI, ischaemia‐reperfusion injury; RLI, regional intra‐arterial limb infusion.

Acute forelimb ischaemia was confirmed grossly via clinical inspection of the forepaw colour turning to pallor upon tourniquet application (Figure 3A). On LSCI flux images, the ischaemic tissues appear as blue (low flux) while normal or hyperperfused regions range from yellow to red colours (high flux) (Figure 3). Similarly, on grey scale images, ischaemic regions exhibit low intensity or black colour whereas normally perfused regions are brighter in colour (Figure 3B, C). Proprietary software analysis of a delineated ROI on flux images provides real‐time perfusion measurements as a.u. In both treatment groups, acute forelimb ischaemia was effectively achieved by tourniquet application at the level of the shoulder joint (Figure 3B, C). Forelimb blood flow was reduced to approximately 74% of the baseline (pre‐ischaemia) values (Figure 3D). This was further validated by the presence of distal forepaw pallor and absence of the triphasic waveform on digital Doppler analysis (Figure 3A).

At 24 h post‐reperfusion and therapeutic delivery using RLI, rats treated with saline‐pEVs and saline‐trehalose demonstrated a 90.77% and 83.41% restoration of blood flow to pre‐occlusion levels (not significantly different). Additionally, no statistical difference was observed between the perfusion units of both treatment groups following RLI and re‐establishment of forelimb blood flow. Overall, the results of these perfusion studies indicated that RLI can be used safely to locally deliver therapeutics such as pEVs to the ischaemic extremity tissue without significant compromise of limb blood flow afterwards.

### 3.4 Xenogen IVIS Imaging and Confocal Microscopy Demonstrate Preferential Uptake and Retention of EVs by Operative Skeletal Muscles Following Regional IA Limb Infusion (RLI) Delivery

**FIGURE 4:**
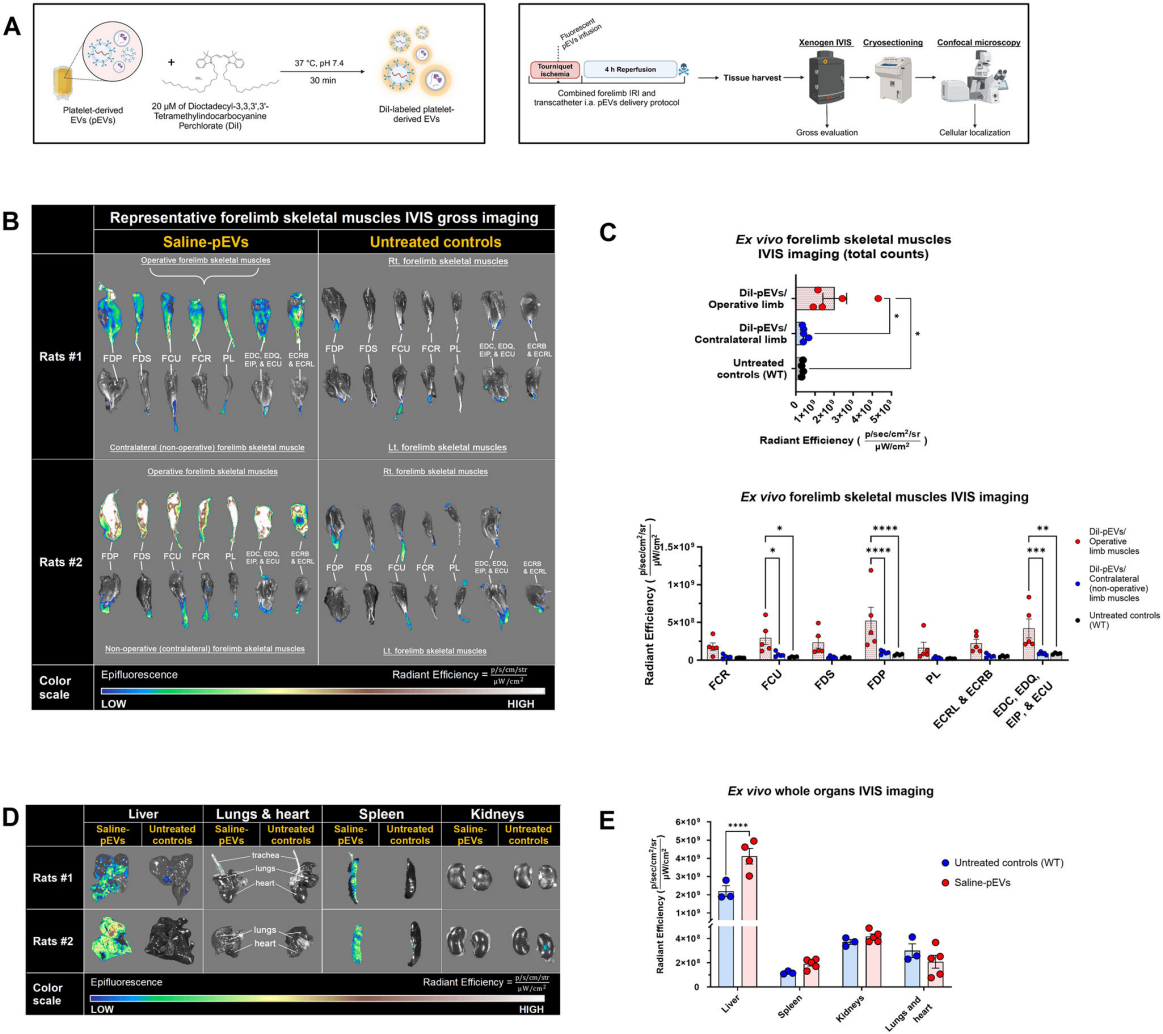
Ex vivo pEVs biodistribution study following transcatheter IA limb infusion delivery. (A) Scheme for pEVs labelling strategy using the fluorescent dye, DiI. (B) Representative gross IVIS imaging of forelimb skeletal muscles from labelled EVs and untreated (WT) controls. The epifluorescence colour scale bar is shown at the bottom of the panel. (C) Quantification of epifluorescence signals from forelimb skeletal muscles. Data presented as mean ± SEM. Statistical comparisons between groups were completed using two‐way ANOVA followed by Tukey's post hoc analysis. *p < 0.05; **p < 0.01; ***p < 0.001; ****p < 0.0001. (D) Representative gross IVIS images of IRI‐injured rats receiving pEVs via RLI versus wild‐type (untreated) subjects. (E) Quantification of epifluorescence signals from major organs of rats receiving pEVs versus WT animals. ANOVA, analysis of variance; EV, extracellular vesicle; IA, intra‐arterial; IRI, ischaemia‐reperfusion injury; RLI, regional intra‐arterial limb infusion; WT, wild‐type.

To evaluate pEVs biodistribution following RLI delivery, forelimb antebrachium skeletal muscles, which are responsible for forepaw function, and major solid organs were immediately dissected at the end of reperfusion and scanned ex situ using the IVIS imaging system. We performed a comparative analysis on age matched healthy SD rats (untreated controls) versus rats that underwent forelimb IRI and received DiI‐labelled pEVs via RLI approach (Figure 4A). Additionally, operative side skeletal muscles were compared to contralateral (non‐operative) forelimb muscles for pEVs uptake in animals receiving treatment (Figure 4B). At the end of the reperfusion phase and RLI delivery, operative forelimb skeletal muscles demonstrated a significant 4.6‐ and 6.03‐fold increase in the total epifluorescence signal compared to the contralateral (non‐surgical) and WT forelimb muscles, respectively (*p* = 0.031) (Figure 4C). Compared to WT forelimbs, muscles of the contralateral (non‐ischaemic) forelimb of DiI‐pEVs treated rats demonstrated a 1.30‐fold change (not statistically different). We also analysed pEVs uptake by individual muscle groups of both forelimbs, which revealed significantly higher radiant efficiency or pEVs fluorescence signals in FDP, FCU and multiple extensor muscles of the forepaw of the operative limb compared to the non‐surgical side and WT forelimb muscles (Figure 4C: bottom). The remainder skeletal muscle groups showed a conspicuous trend although was not statistically significant, probably because of a small sample size. Furthermore, only organs of the RES: liver and spleen of animals that underwent RLI of DiI‐pEVs demonstrated a meaningful change in their fluorescence intensity indicating potential routes of pEVs excretion following this route of EVs delivery (Figure 4D). Essentially, a 1.87‐ (*p* < 0.0001) and 1.62‐ fold change in the epifluorescence intensity of the liver and spleen, respectively, was observed between DiI‐pEVs treated animals versus untreated controls (WT) (Figure 4E).

**FIGURE 5:**
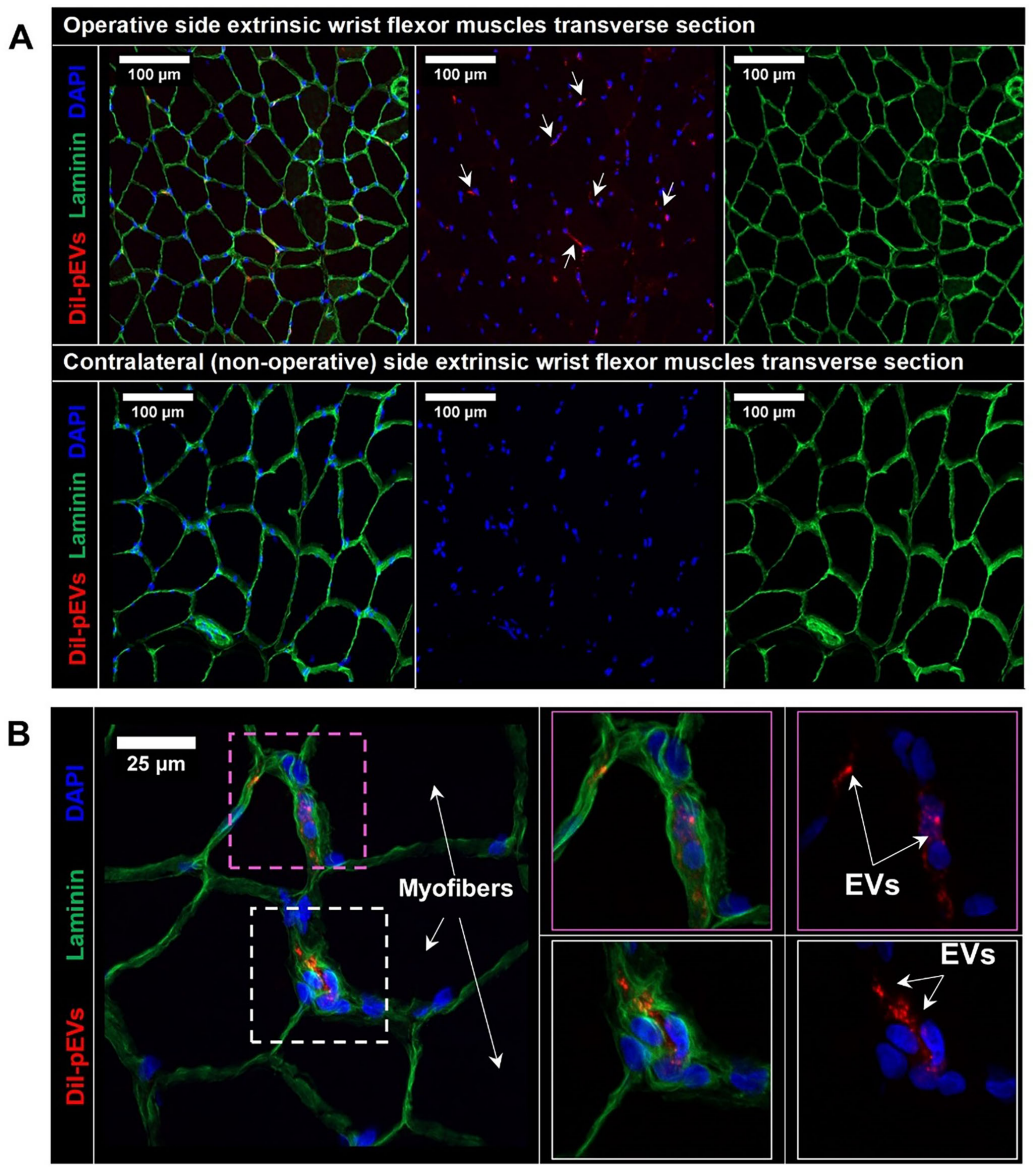
Representative confocal micrographs depicting homing of platelet‐derived EVs into forelimb skeletal muscles following transcatheter IA delivery. Extrinsic wrist flexor muscles (FDP, FDS, FCR, FCU, PL and PT) were harvested en bloc and cryosectioned. Myofibre border demarcation was performed by immunohistochemical staining for laminin (green). (A) Representative immunofluorescent rat forelimb wrist flexor frozen section images from tourniquet IRI model that received EVs via IA delivery approach. Top: operative side forelimb muscles showing DiI‐labelled EVs fluorescence signal mostly from the intermyofibre space and perinuclear regions. Bottom: contralateral (non‐operative) skeletal muscles demonstrating lack of pEVs uptake. (B) High power oil‐immersion lens magnification (x63) visualisation of pEVs uptake by skeletal muscle frozen sections. *n* = 3 rats, all rats received forelimb tourniquet‐induced IRI injury. EV, extracellular vesicle; FCU, flexor carpi ulnaris; FDP, flexor digitorum profundus; IA, intra‐arterial; IRI, ischaemia‐reperfusion injury.

To define the internalisation of pEVs by recipient tissues following RLI delivery, confocal micrographs of muscle tissue transverse sections demonstrated confinement of DiI fluorescence signals in the inter‐myofibre space between the sarcolemma and basal lamina of myofibres of operative side flexor muscles (Figure 5). Furthermore, the use of 63X oil‐immersion objective visualised high concentrations of pEVs aggregates in perinuclear areas of peripheral myofibre nuclei and interspersed cells (Figure 5B). In comparison, contralateral (non‐operative) side flexor muscles lacked detectable DiI fluorescence signal both in inter‐myofibre and perinuclear regions (Figure 5A: bottom panel).

### 3.5 Regional Intra‐Arterial (IA) Limb Infusion of pEVs‐Enriched Preparation Alleviates Metabolic Derangements and Myofibre Injury Induced by Extremity IRI

**FIGURE 6:**
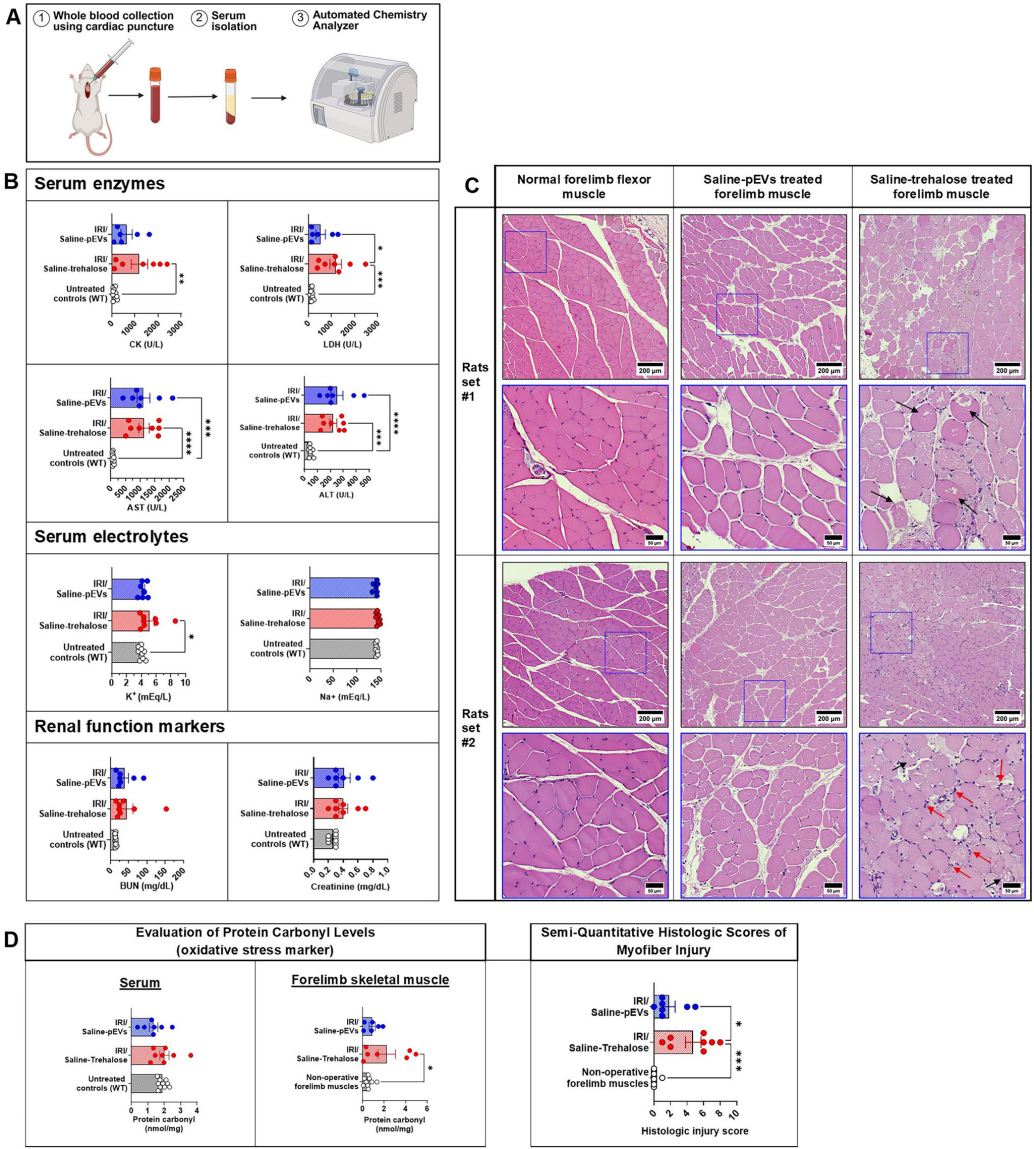
Transcatheter intra‐arterial (IA) delivery of a platelet‐derived EVs (pEVs) enriched preparation mitigates myofibre injury and metabolic derangements associated with peripheral extremity IRI. (A) Schematic for whole blood collection and serum isolation for chemistry analyser processing. (B) Serum chemistry panel: Top: serum enzymes: creatine kinase (CK), lactate dehydrogenase (LDH), aspartate transaminase (AST), alanine transaminase (ALT). Middle: potassium (K^+^); sodium (Na^+^). Bottom: blood urea nitrogen (BUN), creatinine. (C) Top: representative haematoxylin and eosin Y staining of the flexor digitorum profundus muscle (FDP), main extrinsic wrist flexor, from 2 rat sets. Black arrows: sarcoplasmic vacuolations and necrotic, hypercontracted myofibres. Red arrows: immune cell and polymorphonuclear infiltrates. Bottom: evaluation of myofibre injury using semi‐quantitative histologic scoring system. (D) Determination of oxidative stress in serum and skeletal muscle tissue homogenates. Statistical comparisons were performed using one‐way ANOVA followed by Tukey's post‐hoc analysis (*n* = 6–10). *p < 0.05, **p < 0.01, ***p < 0.001, ****p < 0.0001. ANOVA, analysis of variance; EV, extracellular vesicle.

Serum chemistry was used to define the biochemical profiles of animals and assess the magnitude of myonecrosis and organ damage following RLI and reperfusion (Figure 6A, B). Compared to untreated rats, animals that underwent forelimb IRI and received saline‐trehalose IA infusions displayed a significant 10‐fold increase in mean CK levels (*p* = 0.0049), whereas rats treated using saline‐pEVs infusions showed a five‐fold increase in serum CK (*p* = 0.3369). Similarly, serum LDH showed a significant eight‐fold increase in IRI rats compared to WT animals (*p* = 0.0005), showed significantly less LDH levels compared to saline‐trehalose group (*p* = 0.0485). Serum potassium was significantly elevated only in IRI rats receiving saline‐trehalose infusions (*p* = 0.0453).

Both groups demonstrated significantly elevated aminotransferases, ALT and AST, compared to untreated controls (WT) at 24 h following reperfusion and RLI (Figure 6B). Rats treated with saline‐trehalose infusions showed a higher mean BUN (45.00 mg/dL) compared to animals receiving pEVs (38.57 mg/dL); however, there was no statistical difference (Figure 6B). Serum sodium was indifferent among all groups indicating a comparable hydration status in all animals (Figure 6B).

Histopathological assessment of forelimb muscle FFPE sections was performed using a semi‐quantitative scoring system based on inflammatory infiltrate, variation in myocyte shape/size and the number of damaged myofibres per high‐power (40X) field (Figure 6C). Qualitatively, muscle sections from saline‐trehalose‐treated animals frequently displayed sarcoplasmic vacuolations, hypercontracted myofibres, lipid droplet accumulation suggestive of myocyte degeneration and necrosis, and increased immune cell infiltration, consistent with higher histologic injury scores (Figure 6C; black and red arrows). In contrast, saline‐pEV‐treated animals exhibited fewer degenerative and necrotic changes, with relatively preserved myocyte architecture and reduced inflammatory infiltrate (Figure 6C, top).

We modified the standard hypoxic scoring system by excluding oedema, which can be affected by artefactual processing, ensuring that only reliable morphological and cellular criteria were included in the analysis. Compared to non‐operative, contralateral skeletal muscles (mean injury score = 0.11 ± 0.11), forelimb muscles treated with saline‐trehalose demonstrated significantly more severe histologic injury, with a mean score 4.75 ± 0.94, whereas forelimb muscles of rats that received IA infusion of the pEVs‐enriched preparation had lower mean histologic scores of 1.86 ± 0.70, indicating attenuation of tissue injury compared to saline‐trehalose treated animals (Figure 6C, bottom).

Tukey's multiple comparisons test confirmed a highly significant increase in injury scores in the saline‐trehalose‐treated muscles versus non‐operative forelimb muscles (*p* < 0.001). Scores were significantly lower in the saline‐pEVs‐treated forelimb compared to saline‐trehalose (*p* < 0.05) but were not significantly different from control forelimb muscle tissues (Figure 6C, bottom).

Protein carbonyl levels were measured in serum from untreated controls (WT), IRI rats treated with saline‐trehalose and IRI rats treated with pEVs‐enriched formulation (Figure 6D, left). The saline‐trehalose group showed a slightly higher mean (2.01 ± 0.79 nmol/mg) compared to untreated controls (1.89 ± 0.26 nmol/mg), while the animals treated via the pEVs‐enriched preparation had a lower mean (1.35 ± 0.69 nmol/mg). Despite these numerical differences, statistical analysis did not reveal significant differences between groups (*p* > 0.05), indicating that neither saline‐trehalose nor saline‐pEVs treatment produced a measurable effect on systemic oxidative stress, as assessed by serum protein carbonyl content.

Additionally, protein carbonyl content was also quantified in skeletal muscle tissue homogenates from both groups (Figure 6D, right). Operative forelimb skeletal muscles from saline‐trehalose treatment demonstrated a marked increase in mean protein carbonyl levels (2.24 ± 2.15 nmol/mg) compared to contralateral, non‐operative forelimb skeletal muscles (0.55 ± 0.38 nmol/mg). The saline‐pEV group showed a more modest increase (0.89 ± 0.71 nmol/mg), with values closer to baseline. Statistical analysis using one‐way ANOVA followed by Tukey's multiple comparisons test revealed a significant increase in protein carbonyl content in the saline‐trehalose group compared with untreated controls (mean difference = −1.69, 95% CI = −3.33 to −0.046, *p* < 0.05). No significant differences were observed between untreated controls and the saline‐pEVs group or between the saline‐trehalose and saline‐pEVs groups.

These findings indicate that saline‐trehalose treatment significantly increased skeletal muscle oxidative protein damage, whereas saline‐pEVs treatment did not produce a statistically significant effect compared to normal, non‐operative skeletal muscles.

### 3.6 Transcatheter Regional Limb Infusion of a pEVs‐Enriched Formulation Modulates Systemic and Skeletal Muscle Inflammatory Responses Following Ischaemia‐Reperfusion Injury

**FIGURE 7:**
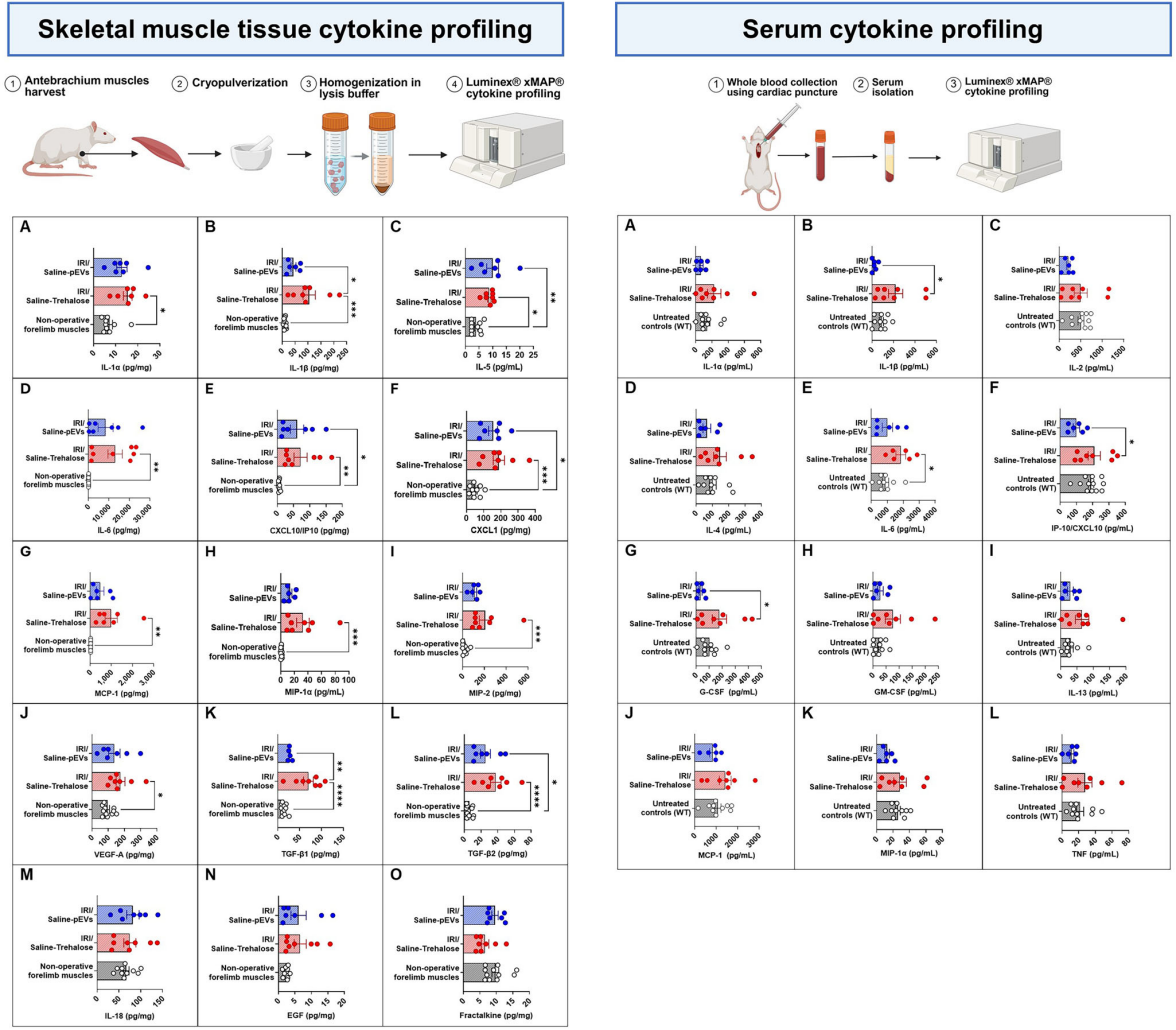
Platelet‐derived EVs suppress cytokine release and monocyte chemoattraction and granulocyte mobilisation following extremity reperfusion injury. Left: forelimb skeletal muscle tissue homogenates cytokine profile: (A) Interleukin 1‐α. (B) Interleukin 1‐β. (C) Interleukin‐5. (D) Interleukin‐6. (E) C‐X‐C motif chemokine ligand 10/interferon gamma‐induced protein 10 (IP‐10). (F) C‐X‐C motif chemokine ligand 1. (G) Monocyte chemoattractant protein‐1 (MCP‐1). (H) Macrophage inflammatory protein‐1 alpha (MIP‐1 α). (I, J) Vascular endothelial growth factor‐A. (K) Transforming growth factor‐β isoform 1. (L) Transforming growth factor‐β isoform 2. (M) Interleukin 18. (N) Epidermal growth factor (EGF). (O) Fractalkine. Statistical comparisons were performed using one‐way ANOVA followed by Tukey's post‐hoc analysis (*n* = 5–11). *p < 0.05, **p < 0.01, ***p < 0.001, ****p < 0.0001. Right: serum cytokine profiling: (A) Interleukin 1‐α. (B) Interleukin 1‐β. (C) Interleukin‐2. (D) Interleukin‐4. (E) Interleukin‐6. (F) C‐X‐C motif chemokine ligand 10/interferon gamma‐induced protein 10 (IP‐10). (G) Granulocyte‐colony stimulating factor. (H) Granulocyte‐macrophage colony‐stimulating factor (GM‐CSF). (I) Interleukin‐13. (J) Monocyte chemoattractant protein‐1 (MCP‐1). (K) Macrophage inflammatory protein‐1 alpha (MIP‐1 α). (L) Tumor necrosis factor (TNF). Statistical comparisons were performed using one‐way ANOVA followed by Tukey's post‐hoc analysis (*n* = 6–10). *p < 0.05, **p < 0.01, ***p < 0.001, ****p < 0.0001. ANOVA, analysis of variance; EV, extracellular vesicle.

We profiled 27 cytokines and chemokines in skeletal muscle tissue homogenates and serum to assess the inflammatory burden following pre‐reperfusion delivery of pEVs‐enriched preparation in our forelimb IRI model (Figure 7). Overall, animals that received pEVs therapy via RLI demonstrated a dampened cytokine response compared to control groups both locally and systemically.

#### 3.6.1 Skeletal‐Muscle Cytokines

Multiplex profiling of muscle lysates revealed a suppressed local cytokine/chemokine response in pEVs‐treated limbs, consistent with inhibition of post‐reperfusion cytokine release (Figure7A–O; left panel). Relative to contralateral normal muscle, IRI + saline‐trehalose treated limbs exhibited significant elevations in IL‐1α (*p* = 0.00122), IL‐1β (*p* = 0.0001), IL‐6 (*p* = 0.0027), VEGF‐A (*p* = 0.0498), MIP‐1α/CCL3 (*p* = 0.0005), MIP‐2/CXCL2 (*p* = 0.0007), CXCL10/IP‐10 (*p* = 0.0069), CXCL1/GROα (*p* = 0.0008), MCP‐1/CCL2 (*p* = 0.0022), TGF‐β1 (*p* < 0.0001) and TGF‐β2 (*p* < 0.0001).

Compared with normal forelimb antebrachium muscles, pEVs‐treated limbs still showed modest increases for MIP‐2/CXCL2, MIP‐1α, CXCL1 (*p* = 0.0137), CXCL10/IP‐10 (*p* = 0.0310) and TGF‐β2 (*p* = 0.0136). More importantly, versus saline‐trehalose, pEVs significantly reduced IL‐1β (*p* = 0.0247) and TGF‐β1 (*p* = 0.0017), with additional percentage reductions in MIP‐1α/CCL3 (58.17% ± 14.61%), MIP‐2/CXCL2 (46.70% ± 16.50%), MCP‐1/CCL2 (52.95% ± 22.69%) and VEGF‐A (21.60% ± 23.30%). Notably, MCP‐1 was largely undetectable in normal muscle (18.2%) but became readily detectable after reperfusion—100% in saline–trehalose and 83.3% in pEVs‐treated limbs indicating group‐dependent expression.

#### 3.6.2 Systemic (Serum) Cytokines

In serum, catheter‐directed delivery of the pEVs‐enriched preparation blunted the IRI‐associated inflammatory response. Relative to IRI/saline–trehalose, pEVs significantly lowered IL‐1β, IL‐6, IP‐10/CXCL10 and G‐CSF (one‐way ANOVA with Tukey's, *p* < 0.05). The largest suppression was seen for G‐CSF (−80.86% ± 37.38%) with substantial decreases in IL‐6 (−45.56% ± 16.45%) and IP‐10/CXCL10; consistent downward trends were also present for MIP‐1α/CCL3 (−55.28% ± 19.14%) and IL‐1α (−70.19% ± 35.14%). IL‐13 fell by −54.47% ± 23.83% versus trehalose and was essentially normalised versus untreated (−2.04% ± 0.83%).

Circulating CXCL1/GROα detection shifted from 62.5% (5/8 of samples analysed) in trehalose and 20% (2/10 of samples analysed) in untreated to 0% (0/6 of samples analysed) in pEVs (two‐sided Fisher's exact, *p* = 0.0310). Collectively, these data indicate reduced granulocyte mobilisation (lower G‐CSF and loss of CXCL1 detection) and diminished monocyte chemoattraction (lower IP‐10/CXCL10 and MIP‐1α/CCL3) with pEVs. Although not all differences reached significance, multiple cytokines (IL‐1α, IL‐2, IL‐4, IL‐13 and TNF‐α) trended towards untreated levels, suggesting near‐normalisation in the pEVs group despite limited sample size.

Across serum and muscle, pEVs administration is associated with a coherent attenuation of post‐IRI inflammation. The paired decreases in G‐CSF, CXCL1/GROα and CXCL2/MIP‐2 support reduced granulocyte (neutrophil) mobilisation, while the coordinated lowering of MCP‐1/CCL2 and MIP‐1α/CCL3 indicates diminished monocyte chemoattraction. These effects accompany significant reductions in canonical pro‐inflammatory cytokines (e.g., IL‐1β and IL‐6), consistent with a broad anti‐inflammatory action of pEVs compared with saline‐trehalose controls.

## 4 Discussion

This study evaluated catheter‐based, IA delivery of allogeneic, off‐the‐shelf human pEV‐enriched preparation in a rodent forelimb skeletal muscle IRI model. In addition to being safe and feasible, pre‐reperfusion delivery of pEVs via an RLI approach achieved effective homing of EVs to target skeletal muscles, mitigated metabolic derangements and suppressed the cytokine storm following extremity reperfusion injury. To our knowledge, this is the first proof‐of‐concept translational study demonstrating efficacy of IA limb perfusion delivery of EVs‐enriched preparations in a small animal model for attenuating extremity IRI, and more exhaustive dosing regimens and optimisation of infusion parameters such as volume and pressure are warranted in future given our preliminary and promising, single‐dose pharmacokinetic results.

Limb preservation strategies for IRI ideally aim to curb the systemic inflammatory response following extremity revascularisation, enhance the ischaemic tolerance of skeletal muscles and nerves via tissue conditioning methods, and prolong the time window from limb amputation to replantation or transplantation (Percival and Rasmussen 2011; Zhang et al. 2024). Inadequate limb preservation or extremely long ischaemia times can trigger lethal metabolic derangements and ‘hypercytokinemia’ that precipitates remote organ injury and deteriorates neuromuscular functionality upon reperfusion. In spite of optimal revascularisation therapy, many patients still suffer from exacerbated myonecrosis, SIRS, haemodynamic instability and multi‐organ failures that could necessitate extremity amputation to control the postreperfusion syndrome (Arango‐Granados et al. 2020; McCutcheon and Hennessy 2002).

Pre‐reperfusion limb preservation techniques are mostly utilised in controlled clinical settings where CPAs can be infused into the limb ex situ before tissue replantation or transplantation (Meyers et al. 2023; Smith et al. 1985). The current gold standard is a ‘preservation flush’ whereby a hypothermic solution such as heparinised saline or acellular preservation fluid, in case of VCA, is continuously infused before storage on ice (cold static storage) (Amin et al. 2018; Kruit et al. 2021). The supplementation of these perfusates with immunomodulatory or anti‐inflammatory biologics such as EVs could potentially maximise limb viability and protect against the SIRS phenomenon following extremity revascularisation. Accordingly, we attempted to mimic pre‐reperfusion limb salvage conditions via ‘in situ’ EVs infusion to the vascularly isolated rodent extremity using a clinically relevant therapeutic delivery paradigm before re‐establishing limb blood flow.

Currently, conventional EVs manufacturing processes are limited by low yields and batch‐to‐batch variability, and hence can obstruct their bench‐bedside translation (Colao et al. 2018; Paganini et al. 2019). Thus, the obtention of EVs produced under cGMP scalable standards can significantly enhance the translational potential of these cell‐free agents. For this drug delivery feasibility study, we used an externally sourced, off‐the‐shelf pEVs‐enriched formulation as a surrogate EVs test article to evaluate our transcatheter RLI approach in the rat forelimb. Based on our TEM and NTA data, cGMP‐compliant pEVs‐enriched formulations used herein demonstrated relative lot‐to‐lot manufacturing consistency and EVs homogeneity from the concentration, morphological, size and cargo content perspectives. Essentially, pEVs used in this study comprised mostly of small EVs (sEVs) or EVs measuring less than 200 nm in diameter according to the MISEV2023 guidelines and expressed canonical EV markers. This data further reinforces pEVs characterisation results from previous results utilising a similar platform (Rolland et al. 2022; Colao et al. 2018; Shi et al. 2021).

Furthermore, small‐RNA sequencing revealed an miRNA signature consistent with pEVs, supporting the use of a platelet derivative. In line with our findings, canonical platelet miRNAs like miR‐223‐3‐3p, miR‐126‐3p/‐5p, miR‐21‐5p, miR‐423‐5p and miR‐146 have been reported by other groups to be abundant in pEVs with anti‐inflammatory and immunomodulatory functions (Table S2). Additionally, multiplexed protein profiling of bulk EVs lysates detected CD9, CD63 and TSG101, but not CD81 or flotillin, which confirmed EVs identity. A similar report utilising the same pEVs similarly documented CD9 detection on multiplex immunoassay but not CD81 and CD63 (Beetler et al. 2024).

However, detectable levels of the ER‐derived protein, CALR, were identified highlighting contamination by other non‐vesicular cellular components. Additionally, the same proprietary, off‐the‐shelf, EVs‐based technology was reported to also contain apolipoprotein A1 (ApoA1), which is a common contaminant of plasma/platelet EV isolation workflows (Beetler et al. 2024). Notably, the field currently lacks validated thresholds/cutoffs for purity metrics or consensus ‘benchmark’ values that would allow categorical classification of a given preparation as ‘pure’. Purification of plasma‐derived EVs is particularly challenging because pEVs and lipoproteins overlap in size and density, rendering high‐purity isolation problematic despite ongoing efforts to enhance EVs compositional purity (Chou et al. 2024; Kapoor et al. 2024).

More broadly, these findings motivate rigorous, transparent QC reporting for pEV products, particularly as EV‐based therapeutics advance towards commercialisation and regulatory evaluation. Nevertheless, to reflect this heterogeneous composition of the off‐the‐shelf, lyophilised preparation used herein, we deliberately refer to the material used as an EVs‐enriched formulation rather than a highly purified EVs product.

Furthermore, EVs compositional data are important for interpreting in vivo functional outcomes and bioactivity. Although multi‐modal profiling of the externally sourced EVs preparation used in this study supports EVs enrichment, we cannot fully exclude contributions from co‐isolated non‐vesicular species (e.g., lipoproteins) to the anti‐inflammatory signal observed; definitive attribution of bioactivity to EV cargo will require fractionation and vesicle‐disruption/immunodepletion controls in future studies. For instance, acute exposure to ApoA1 has been reported to modulate monocyte/macrophage trafficking and exert anti‐inflammatory effects, which could act alongside—or independent of—EV cargo (Hyka et al. 2001; Iqbal et al. 2016). All animals, however, received identically prepared, lyophilised pEVs‐enriched lots, controlling for between‐group manufacturing variability. Thus, while we underscore the importance of purity and transparent composition, this study was designed as a proof‐of‐concept to demonstrate the feasibility of transcatheter IA EVs delivery to the rodent forelimb and its capacity to mitigate skeletal muscle ischaemia–reperfusion injury in vivo. Limb perfusion delivery of therapeutics has been utilised for administering adenoviral vectors, cytotoxic chemotherapy for sarcomas and endovascular thrombolytics (Parker et al. 2023; Schrijver et al. 2015; Neuwirth et al. 2017). Recently, the IA or intracoronary route of delivering EVs to tissues has also been leveraged to reduce infarct size and protect the myocardium in translational models of acute myocardial infarction and reperfusion injury (Wang et al. 2021; Emmert et al. 2024). Also, few reports have attempted IA delivery to expand EVs homing capabilities to target other tissues including the kidneys and pancreas (Ullah et al. 2020; Li et al. 2022).

Our results in this study showed that a catheter‐based IA infusion protocol can be harnessed to enhance local EVs delivery to the ischaemic extremity and overcome off‐target accumulation. Rapid clearance of EVs from the systemic circulation poses a tremendous challenge for using systemic routes of administrations for EVs delivery. Majority of administered nanomaterials including EVs are sequestered by macrophages of the RES and preferentially accumulate in RES organs such as the liver and spleen (Tsoi et al. 2016; Choi and Lee 2016; Kang et al. 2021). For instance, Haga et al. (2017) showed a seven‐fold increase in the epifluorescence signal of both the liver and spleen 6 h following IV delivery of bone marrow MSCs‐derived EVs in a mouse model of hepatic I/R injury compared to untreated controls. Although we observed an increase in ex vivo hepatic and splenic fluorescence signals following DiI‐pEVs administration, the fold‐difference between locally treated skeletal muscles and their controls significantly surpassed the fold‐change in fluorescence intensity between treated and untreated solid organs, suggesting effective localisation of pEVs to the treated forelimb. Despite IVIS evidence suggesting minimal systemic spillover, our study is limited by the lack of a head‐to‐head comparison of IA, IV and IM delivery, which precludes definitive quantification of the IA route's regional selectivity and skeletal‐muscle retention. However, given that the contralateral (non‐treated) skeletal muscles of animals receiving DiI‐pEVs were not statistically different compared to untreated subjects, we can infer that using routes such as IV for EVs delivery would ultimately result in substantially minimal uptake by skeletal muscles for the same pEVs dose due to first pass effect or necessitate exorbitant doses to achieve therapeutic efficacy.

We then attempted to define alterations of serum biomarkers following EVs delivery in our tourniquet I/R injury model at 24 h post‐limb reperfusion. Muscle enzymes typically surge to several folds the normal values in states of extensive sarcolemmal disruption and necrosis that generally occurs following revascularisation of myocardial or peripheral extremity skeletal muscles previously subjected to prolonged ischaemia (Keltz et al. 2013; Kodadek et al. 2022). Furthermore, CK and LDH values can serve as prognostic indicators in rhabdomyolysis‐induced acute kidney injury in patients with postreperfusion syndrome (Nielsen et al. 2020; Heidari Beigvand et al. 2021). Here, we demonstrated that a single‐dose of pEVs delivered before reestablishing rat forelimb blood flow resulted in a diminution of CK and LDH values, suggesting a potential myoprotective effect of pEVs in extremity IRI. Cui et al. (2017) similarly reported a significant decline in serum CK and LDH values following tail vein infusion of adipose‐derived MSCs‐EVs in a rat model of myocardial IRI within 5 min of the onset of reperfusion. Furthermore, Luo et al. (2023) perfused isolated rat hearts with plasma EVs obtained following cardiac ischaemic‐preconditioning (IPC) stimulus and showed their cardioprotective potential against cardiac I/R injury in both normal and failing hearts. Essentially, IPC‐derived EVs significantly reduced both LDH levels in coronary effluents and infarct size percentage on triphenyltetrazolium chloride staining following reperfusion in their ex vivo global I/R injury model.

Moreover, our data showed the potential of pEVs in suppressing cytokine storm and ligands implicated in monocyte chemotaxis and neutrophil mobilisation that typically occur following extremity reperfusion injury (Kim et al. 2024). Several reports have documented the intrinsic capacity of EVs isolated from different cellular origins to neutralise the aberrant cytokine release secondary to various pathologies including COVID‐19, sepsis and status epilepticus (Ge et al. 2024; Wang et al. 2022; Liu et al. 2024). However, available studies on pEVs described their dual roles in inflammatory conditions, exhibiting either pro‐ or anti‐inflammatory effects depending on their surface markers expression and cargo (Jiang et al. 2023; Vajen et al. 2017; Zhu et al. 2023). Nevertheless, few studies documented pEVs’ ability to suppress systemic inflammation by primarily inhibiting cytokine release as we have noted in this study (Xuan et al. 2024; Ma et al. 2020).

At 24 h following forelimb I/R, IA administration of our EV‐enriched preparation was associated with a selective attenuation of monocyte‐associated inflammatory signalling, evidenced by lower MCP‐1/CCL2, IL‐1β, IL‐6, CCL3/MIP‐1α and TGF‐β compared with the control treatment, whereas neutrophil‐linked chemokines (CXCL1 and CXCL10) remained elevated in both treatment groups relative to untreated animals. This pattern suggests that, at this timepoint, a preferential modulation of the CCR2/monocyte axis occurs rather than broadly suppressing inflammation or fully dampening the early CXCR2/neutrophil chemokine program.

Although we could not establish causation for this immunomodulatory profile, small‐RNA sequencing of the EV‐enriched formulation delivered revealed abundance of miR‐146a‐50, miR‐223‐3p, miR‐16‐5p, let‐7 family and miR‐126, miRNAs previously linked to dampening TLR (Toll‐like receptor)/NF‐κB–inflammasome signalling and endothelial activation, providing biological plausibility for the observed cytokine response (Table S2) (Zhou et al. 2018; Xi et al. 2022; Ye and Steinle 2016; Jimenez Calvente et al. 2020). These associations do not establish causality, and, while the data are consistent with reduced CCR2/monocyte‐driven amplification at this timepoint, effects on neutrophil recruitment remain to be determined. The concurrent reduction in VEGF in EV‐treated limbs versus control further supports lower endothelial stress/permeability at 24 h. Collectively, these results indicate an early myoprotective profile following the IA limb delivery of an EV‐enriched material—selectively curbing pro‐inflammatory and pro‐fibrotic mediators while preserving early chemotactic tone, and align with our aim of establishing the feasibility and potential benefit of locoregional EV‐based therapy for extremity reperfusion injury.

Beetler et al. (2024) also documented the specific targeting of the TLR and inflammasome pathways contributing to murine viral myocarditis by pEVs, which were further documented using microRNA and proteomic sequencing. These findings, overall, supports our preliminary hypothesis that pEVs‐mediated immunomodulatory and inflammation suppressing roles may involve direct effects on TLR inflammasome signalling. However, more rigorous mechanistic research is vital to establishing direct causal links between the pEVs cargo and the immunomodulatory effects observed here and not by the co‐isolated non‐vesicular contaminants. As highlighted throughout the text, a major limitation of this study is the use of an externally sourced EVs‐enriched product that lacks lot‐specific quality control data. Although our in‐house characterisation confirmed the presence of canonical EV markers, it also revealed contamination with intracellular, non‐vesicular proteins, such as CALR. Future work will involve the use of MISEV‐compliant pEVs to validate these findings and dissect EVs‐specific roles in myoprotection and neuromuscular recovery following the extremity post‐reperfusion syndrome.

Moreover, multiple parameters were not optimised for limb infusion delivery and dosage selection of the off‐the‐shelf pEVs‐enriched preparation used herein, which are imperative for determining maximal therapeutic efficacy. Second, determining the optimal therapeutic window for pEVs administration, whether during pre‐ischaemia, pre‐reperfusion or post‐reperfusion phases, has not been investigated in this study and needs to be addressed in future work. Third, given the translational focus of our study, we did not report mechanistic experiments to delineate putative pathways or pEVs mediators implicated in sarcolemma stability and oncosis resistance, which are beyond the scope of this article. Nonetheless, the manifest anti‐inflammatory effects of the pEVs‐enriched formulation demonstrated by our data, both locally and systemically, could partially explain the reduced myofibre damage and improved serum biochemical profiles observed in rats treated with IA delivery of the pEVs‐enriched preparation. Lastly, the short‐term follow‐up durations chosen for this study are based primarily on the hyperacute nature of skeletal muscle IRI. Additional investigation of the long‐term therapeutic effects of pEVs administration is required using I/R injury models of prolonged limb ischaemia and well‐designed limb preservation regimens.

In summary, we propose an innovative strategy for pre‐reperfusion limb preservation using catheter‐directed IA infusion of pEVs‐enriched formulations to curb the metabolic alterations and hyperinflammatory state resulting from extremity postreperfusion syndrome. In the locally treated limb, pEVs were distributed homogenously following a single, IA infusion dose and mean total uptake of EVs by skeletal muscles exceeded four‐ and six‐folds that of the contralateral limb and untreated animals, respectively. Our research findings have broad implications across multiple fields where extremity postreperfusion syndrome presents both research and clinical challenges, offering valuable insights into novel approaches for limb salvage.

## Author Contributions

**Omar A. Selim**: conceptualisation, writing – original draft, writing – review and editing, visualisation, methodology, software, data analysis and curation, investigation, validation, funding acquisition. **Aida Sarcon**: data analysis and curation. **Atta Behfar**: resources. **Chunfeng Zhao**: data analysis, investigation, validation, resources. **Steven L. Moran**: conceptualisation, writing – review and editing, validation, methodology, supervision, resources, funding acquisition.

## Conflicts of Interest

Atta Behfar is the founder and on the board of directors for Rion, Inc. Steven L. Moran is on the advisory board for Rion, Inc. Omar A. Selim has been funded by NIH T32 AR56950 training grant while conducting this study. The rest of the authors have no financial disclosures.

## Supporting information




**Supporting Information Figure S1**: jev270247‐sup‐0001‐FigureS1.docx

**Supporting Information Table S1–S2**: jev270247‐sup‐0002‐TableS1‐S2.docx

## Data Availability

The data that support the findings of this study are available from the corresponding author upon reasonable request.
