# Supplementary material for: Transcatheter Intra‐Arterial Delivery of a Platelet‐Derived Extracellular Vesicle‐Enriched Preparation for Attenuating Skeletal Muscle Ischaemia‐Reperfusion Injury in a Rodent Forelimb Model
Source: J Extracell Vesicles. 2026 Apr 17;15(4):e70247. doi: 10.1002/jev2.70247 (PMC13088881; doi:10.1002/jev2.70247)
Supplement: Supplementary file 1 — Supporting Information Figure S1: jev270247‐sup‐0001‐FigureS1.docx [file JEV2-15-e70247-s001.docx]

**Supplementary Figure 1**

**Rat 2 Native forelimb angiogram & DSA image sequence**

**Rat 1 DSA image sequence**

| 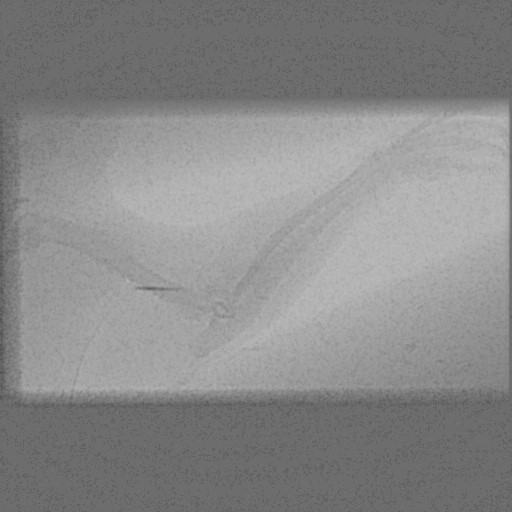 |
| --- |
| 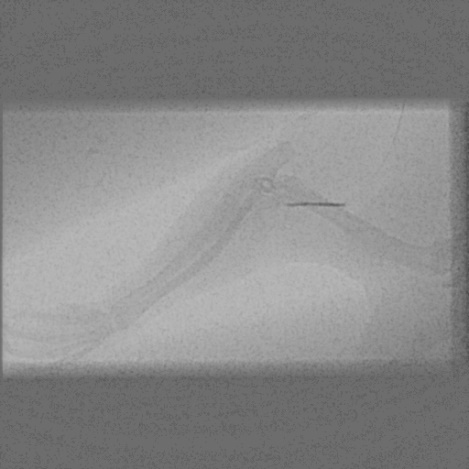 |
| 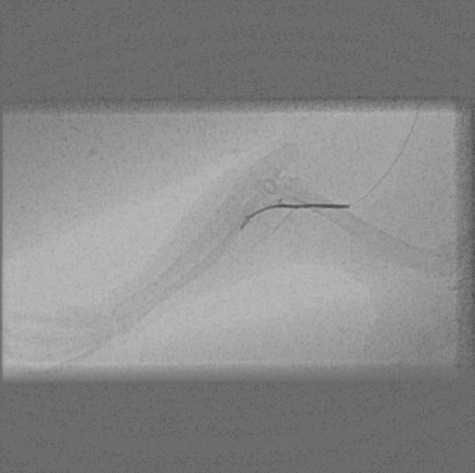 |
| 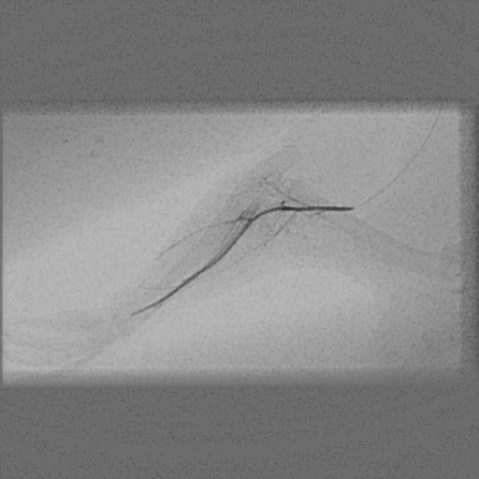 |
| 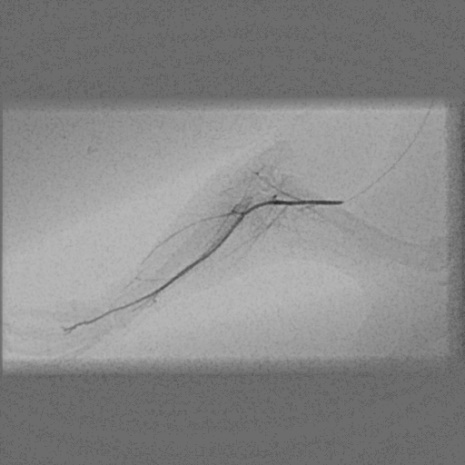 |
| 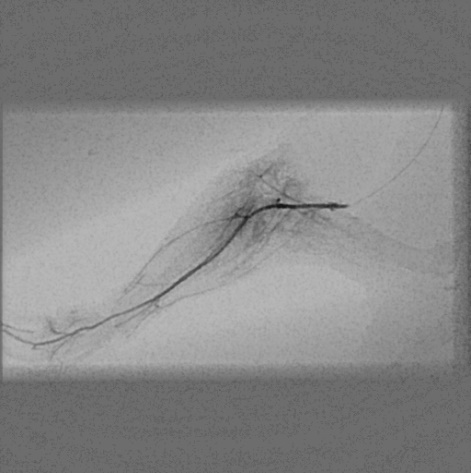 |

| 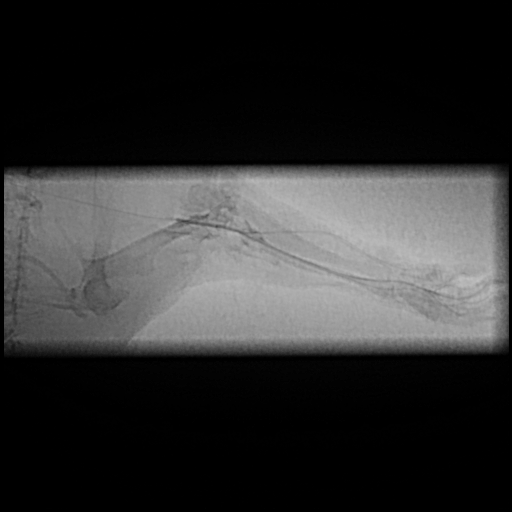 |
| --- |
| 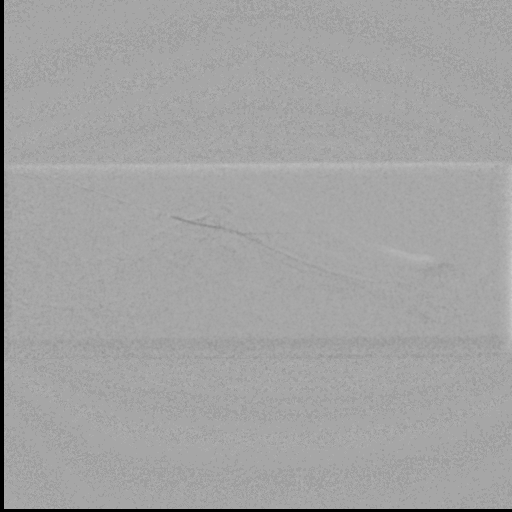 |
| 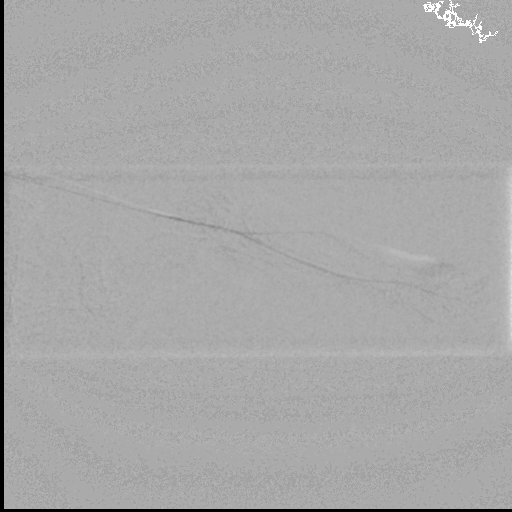 |
| 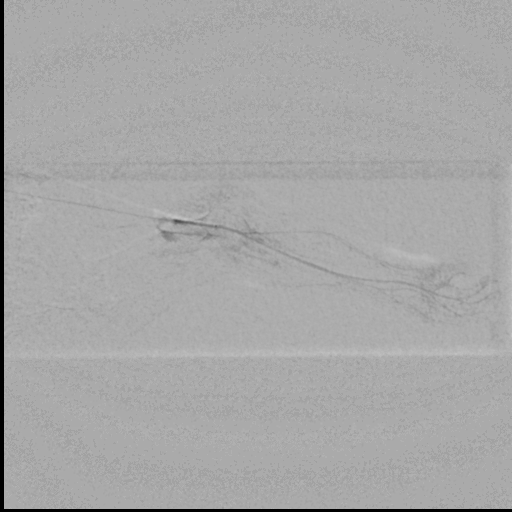 |

**3**

**2**

**1**

**6**

**5**

**4**

**3**

**2**

**1**
