# Supplementary material for: Transcatheter Intra‐Arterial Delivery of a Platelet‐Derived Extracellular Vesicle‐Enriched Preparation for Attenuating Skeletal Muscle Ischaemia‐Reperfusion Injury in a Rodent Forelimb Model
Source: J Extracell Vesicles. 2026 Apr 17;15(4):e70247. doi: 10.1002/jev2.70247 (PMC13088881; doi:10.1002/jev2.70247)
Supplement: Supplementary file 2 — Supporting Information Table S1–S2: jev270247‐sup‐0002‐TableS1‐S2.docx [file JEV2-15-e70247-s002.docx]

**Supplementary Table 1: MISEV2023-guided protein profiling of an off-the-shelf lyophilized platelet-derived EV formulation.**

| **MISEV2023 Category** | | **Marker** | ***n*** | **Mean (pg/mg) ± SD** | **Range**  **(pg/mg)** | **CV%** |
| --- | --- | --- | --- | --- | --- | --- |
| **Category 1 –** Transmembrane (or GPI-anchored) proteins associated with plasma membrane and/or endosomes | **1a:** Multi-pass transmembrane proteins. | CD63 | 4 | 136.02 ± 12.90 | 122.46 - 153.23 | 9.48 |
|  |  | CD9 | 4 | 1. ± 138.50 | 156 - 494 | 43.82 |
|  |  | CD81 | 4 | OOR (all vials) | - | - |
| **Category 2 –** Cytosolic proteins in EVs | **2a:** ESCRT-I/II/III and accessories | TSG101 | 4 | 119.64 ± 41.04 | 78.778 - 173.14 | 34.31 |
|  |  | Flotillin | 4 | OOR (all vials) | - | - |
|  |  | Syntenin | 4 | OOR (all vials) | - | - |
|  | **2b:** promiscuous  incorporation into EVs | GAPDH | 4 | 2448.89 ± 63.63 | 2387.47 - 2537.79 | 2.60 |
| **Category 4 –** Transmembrane, lipid-bound and soluble proteins associated with intracellular compartments other than PM/endosomes | **4c:** secretory pathway.  Endoplasmic reticulum | Calreticulin | 4 | 313.702 ± 140.92 | 189.70 - 504.82 | 44.92 |
| **Category 5 –** Secreted proteins recovered with EVs: Corona or functional component of EVs | **5b:** cytokines and  growth factors. | TGF-β1 | 4 | 176.81 ± 9.93 | 163.40 - 186.99 | 5.62 |
|  |  | TGF-β2 | 4 | 12.19 ± 1.47 | 10.54 - 14.08 | 12.06 |
|  |  | TGF-β3 | 4 | 0.54 ± 0.11 | 0.45 - 0.70 | 20.72 |
|  |  | Ang-2 | 4 | 5.74 ± 1.84 | 4.06 - 8.29 | 32.08 |
|  |  | BMP9 | 4 | 2.42 ± 0.18 | 2.21 - 2.60 | 7.54 |
|  |  | EGF | 4 | 9.41 ± 1.25 | 8.21 - 10.50 | 13.32 |
|  |  | Endoglin | 4 | 16.72 ± 2.91 | 13.70 - 20.14 | 17.43 |
|  |  | FGF-2 | 4 | 8.13 ± 3.77 | 4.57 - 13.23 | 46.31 |
|  |  | Follistatin | 4 | 18.60 ± 1.13 | 17.35 - 20.05 | 6.08 |
|  |  | HB-EGF | 4 | 4.22 ± 0.48 | 3.77 - 4.83 | 11.33 |
|  |  | HGF | 4 | 4.90 ± 2.21 | 2.53 - 6.78 | 45.00 |
|  |  | Leptin | 4 | 515.86 ± 65.31 | 441.31 - 584.80 | 12.66 |
|  |  | VEGF-A | 4 | 9.31± 3.04 | 6.83 - 13.69 | 32.59 |
|  |  | VEGF-C | 4 | 60.63 ± 8.54 | 52.59 - 68.75 | 14.08 |
|  |  | VEGF-D | 4 | 2.38 ± 0.80 | 1.71 - 3.46 | 33.57 |
| ESCRT = Endosomal Sorting Complex Required for Transport; GAPDH = Glyceraldehyde-3-phosphate dehydrogenase; ApoA1/ApoB = Apolipoproteins A1/B; TGF = Transforming Growth Factor; Ang-2 = Angiopoietin-2; EGF = Epidermal Growth Factor; FGF-1/2 = Fibroblast Growth Factor-1/2; G-CSF/CSF-3 = Granulocyte Colony-Stimulating Factor; HB-EGF = Heparin-binding EGF-like Growth Factor; HGF = Hepatocyte Growth Factor; IL-8 (CXCL8); PlGF = Placental Growth Factor; VEGF-A/C/D = Vascular Endothelial Growth Factors A/C/D. | | | | | | |

**Supplementary Table 2: Literature-mined evidence for platelet(-EV) miRNAs detected in the pEVs-enriched formulation used in the study and their reported roles in inflammation.**

|  | **Documented as a platelet-EVs miRNA** | **Role in inflammation** | **Pathway/targets** | **References** |
| --- | --- | --- | --- | --- |
| **hsa-miR-223-3p** | PMID: 35132909.  PMID: 23323973.  PMID: 23652806.  PMID: 34135769. | Anti‑inflammatory | Direct repression of NLRP3 → ↓inflammasome activation; dampens TLR/NF‑κB cytokines | PMID: 22984082.  PMID: 31585800.  PMID: 38448875. |
| **hsa-miR-126-3p / -5p** | PMID: 23323973.  PMID: 23386708. | Anti‑inflammatory | Endothelial VCAM‑1 repression; attenuates leukocyte adhesion and NF‑κB programs | PMID: 18227515.  PMID: 22419694. |
| **hsa-miR-21-5p** | PMID: 23323973. | Anti‑inflammatory (context‑dependent) | Targets PDCD4 → ↑IL‑10; limits NF‑κB activation in macrophages/vascular cells | PMID: 24391209.  PMID: 38317820. |
| **hsa-let-7 family** | PMID: 23226537. | Often anti‑inflammatory (context‑dependent) | let‑7b/i target TLR4 → ↓NF‑κB/cytokines; some pro‑allergic roles reported | PMID: 33868293.  PMID: 37155468. |
| **hsa-miR-16-5p** | PMID: 32347048.  PMID: 31269049 | Anti‑inflammatory | Targets TLR4 → inhibits TLR4/NF‑κB; protects epithelial barrier in inflammatory models | PMID: 36067883. |
| **hsa-miR-423-5p** | PMID: 23493781.  PMID: 23226537. | Often anti‑inflammatory (context‑dependent) | Modulates NF‑κB via NLRX1 axis (disc models); stimulus‑responsive release from platelets | PMID: 32467560. |
| **hsa-miR-151a-3p** | PMID: 35455934. | Mixed (pro in osteoclastogenesis; anti‑apoptotic in SCI models) | Promotes osteoclast differentiation; exosomal miR‑151‑3p attenuates apoptosis via p53/p21/CDK1 | PMID: 34290498.  PMID: 35127706. |
| **hsa-miR-191a-3p** | PMID: 35455934. | Pro‑inflammatory (endothelial/EV contexts) | Activates NF‑κB signaling; EV‑miR‑191‑5p induces NF‑κB and IL‑8 in recipient cells | PMID: 28424351.  PMID: 39199417. |
| **hsa-miR-146a-5p** | PMID: 24908639.  PMID: 21364189. | Anti‑inflammatory (canonical) | Targets IRAK1/TRAF6 → negative feedback on TLR‑NF‑κB; protects in multiple inflammation models | PMID: 16885212.  PMID: 21576471.  PMID: 24987958. |
| **hsa-miR-432-5p** | **-** | Anti‑inflammatory (chemokine axis) | Targets CXCL5 (pro‑inflammatory chemokine) → ↓chemotaxis/inflammatory signaling (CRC models) | PMID:33717244. |
| **hsa-miR-148a-3p** | **-** | Anti‑inflammatory | Represses TLR4/MyD88/IRAK1/TRAF6 → ↓NF‑κB; can also modulate PTEN→AKT1 axis | PMID: 31756048.  PMID: 28646187. |
